# Geoglyphs and formative-period activity in the middle Chillón Valley, Peru: Ceramic association and null-model tests of route proximity

**DOI:** 10.1371/journal.pone.0350855

**Published:** 2026-06-08

**Authors:** Christian Mesía-Montenegro, Ángel Sánchez-Borjas

**Affiliations:** 1 Universidad Privada del Norte, Dirección de Investigación, Innovación y Sostenibilidad,‌‌ Lima, Peru; 2 Pontificia Universidad Católica del Perú, Programa de Estudios Andinos, ‌‌Lima, Peru; German Archaeological Institute: Deutsches Archaologisches Institut, GERMANY

## Abstract

Geoglyphs are widespread in arid landscapes, but their chronology and relationship to movement corridors are often difficult to evaluate where direct dating is unavailable. This study examines four geoglyph groups documented through systematic pedestrian survey and RPAS-based recording in two quebradas of the middle Chillón Valley, central coast of Peru: Huarabí (n = 2) and Pichausa (n = 2). Chronological inference is necessarily limited. Diagnostic Formative-period ceramics were recorded only in Huarabí, where they indicate nearby activity rather than directly dating geoglyph construction. Pichausa remains chronologically unresolved. To evaluate whether the documented geoglyphs occur closer to mapped route proxies than expected under explicit counterfactual assumptions, we measured nearest-route distances from within-geoglyph sampling points (k = 20 per geoglyph; n = 80 total) and from six unweighted Formative ceramic findspots in Huarabí. Observed distance distributions were compared with Monte Carlo simulations in which geoglyph shapes were randomly repositioned within defined survey windows while route geometries remained fixed. Across 999 simulations, Huarabí deviates from the tested null model under the survey-polygon window, or buffer = 0 m (p = 0.021), whereas Pichausa does not (p = 0.380). Results vary substantially under broader availability windows, indicating that inference is strongly conditioned by how potential placement space is defined.. These findings provide exploratory, model-dependent evidence that some Huarabí geoglyph contexts and associated ceramic findspots occur nearer to mapped route proxies than expected under the specified null model. They do not establish intentional route association, geoglyph construction age, or valley-wide spatial organization. The principal contribution is methodological: the study demonstrates how small geoglyph datasets can be evaluated through transparent null-model procedures while making explicit the limits imposed by sample size, surface chronology, spatial uncertainty, and the absence of terrain-based feasibility modeling.

## Introduction

**Fig 1:**
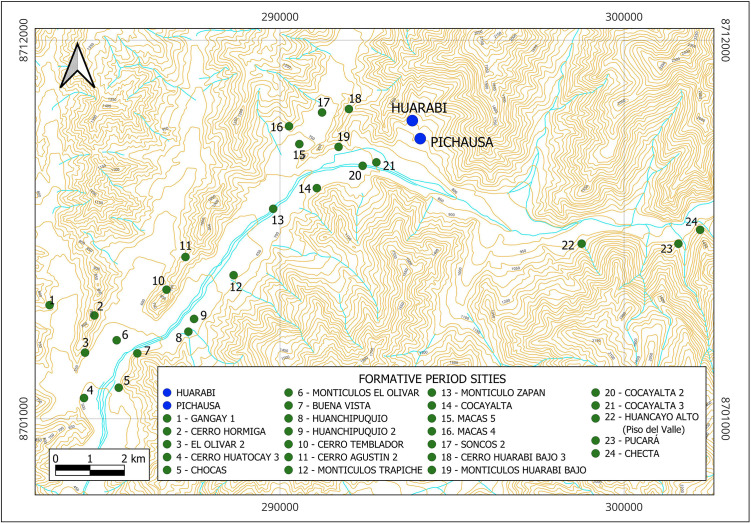
Chillón drainage and mapped archaeological features. Topographic map of the Chillón drainage, central coast of Peru, showing the locations of the geoglyph localities examined in this study in relation to selected archaeological sites. Huarabí and Pichausa are marked as geoglyph localities. Red symbols indicate U-shaped buildings, including El Paraíso, Infantas, Chuquitanta A, Chuquitanta B, Huacoy, Chocas, and Pucará. Buena Vista is shown separately as a monumental site not classified here as a U-shaped building. The inset locates the study area within Peru. Base cartography: Carta Nacional Vectorial del Perú, Instituto Geográfico Nacional del Perú; ‌‌cartographic processing in QGIS. Archaeological data are author-generated field/GIS data.

**Fig 2:**
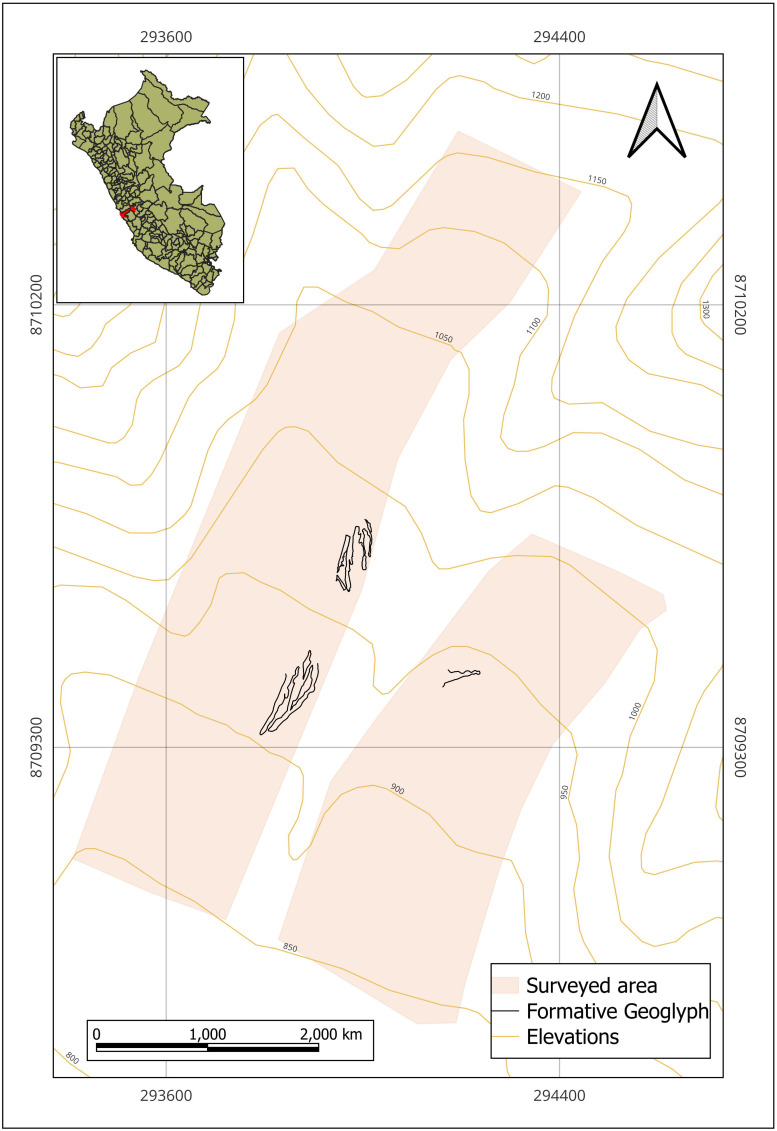
Huarabí survey sectors and mapped Formative geoglyphs. Detail map of the Huarabí sector showing surveyed areas, contour lines, and mapped Formative geoglyph linework. The shaded polygons delimit the surveyed areas used to define the local spatial context for geoglyph documentation and proximity analysis. Geoglyph linework is shown in black. The inset locates the mapped sector within Peru. Base cartography: Carta Nacional Vectorial del Perú, Instituto Geográfico Nacional del Perú; cartographic processing in QGIS. Geoglyph and survey-area data are author-generated field/GIS data.

**Fig 3:**
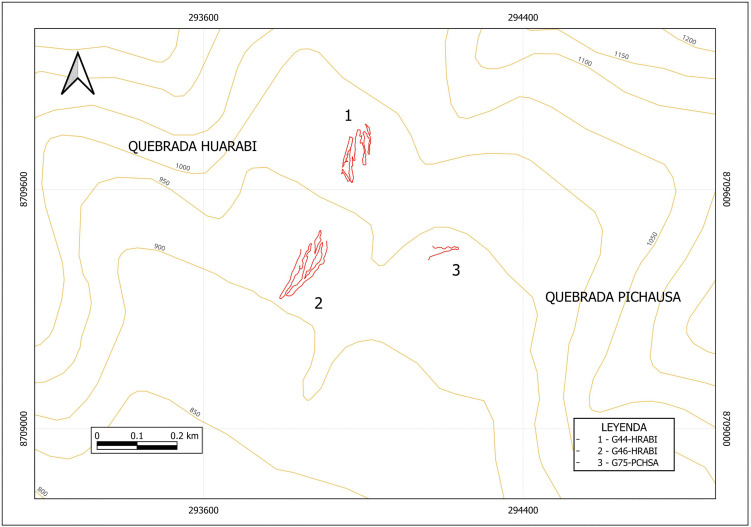
Geoglyph groups documented in the Huarabí and Pichausa sectors. Map showing the three geoglyph groups used in the comparative spatial analysis: G44-Huarabí, G46-Huarabí, and G75-Pichausa. The figure shows their relative positions within the two quebrada settings, with contour lines indicating local terrain morphology. Labels identify the Huarabí and Pichausa sectors. Base cartography: Carta Nacional Vectorial del Perú, Instituto Geográfico Nacional del Perú; cartographic processing in QGIS. Geoglyph data are author-generated field/GIS data.

**Fig 4:**
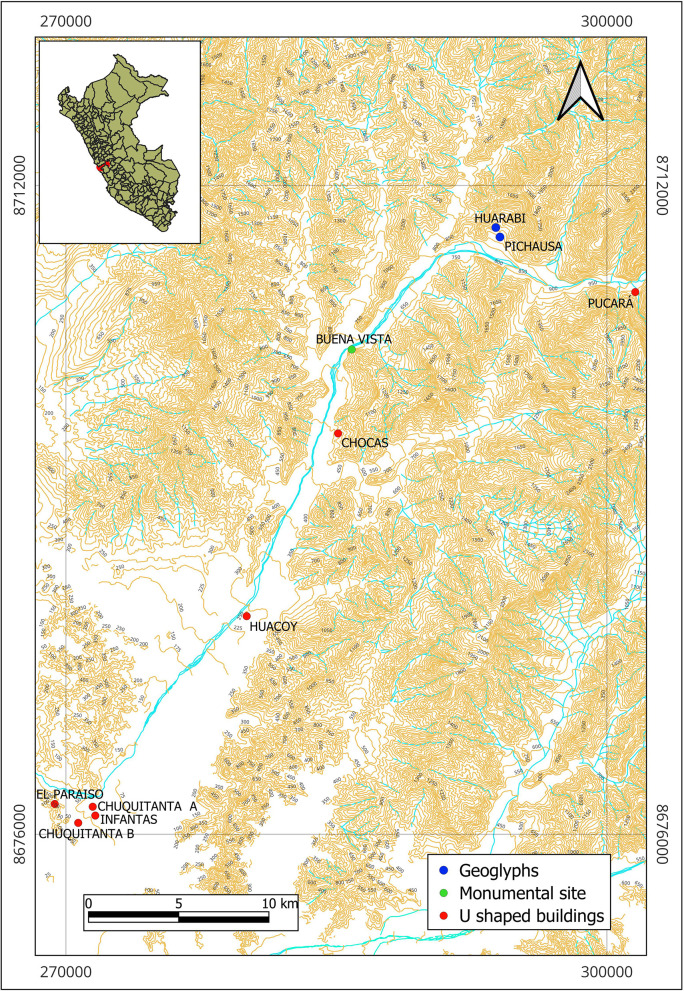
Formative-period archaeological sites in the middle Chillón drainage. Map of Formative-period sites and geoglyph localities in the middle Chillón drainage. Huarabí and Pichausa are highlighted as the geoglyph localities examined in this study. Numbered green symbols indicate Formative-period sites used for regional contextualization. Base cartography: Carta Nacional Vectorial del Perú, Instituto Geográfico Nacional del Perú; cartographic processing in QGIS. Archaeological site locations were compiled from author field records.

**Fig 5:**
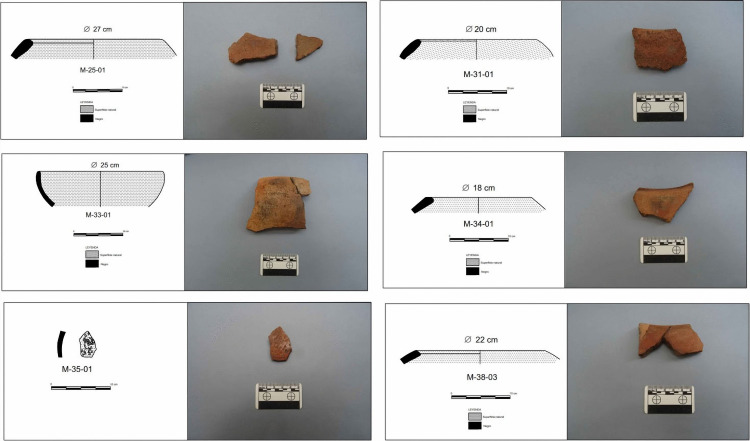
Diagnostic Formative ceramics from Huarabí surface contexts adjacent to geoglyph locales. Illustrated and photographed ceramic sherds from Huarabí used for the study’s relative chronological assessment. The sample includes diagnostic rim fragments and one decorated body sherd recorded in surface contexts near the Huarabí geoglyphs. These materials provide evidence of Formative-period activity in the vicinity of one geoglyph locality, but they do not directly date geoglyph construction. Scale bars in centimeters.

A previous paper by the authors presented the broader survey project from which this dataset derives [1]. That article provided a regional overview of the fieldwork and documentation. The present paper has a narrower aim, it focuses on geoglyphs from Huarabí and Pichausa and on the Formative ceramic findspots recorded near the Huarabí linework (Figs 1–5). Rather than repeating the earlier survey overview, it evaluates whether the documented geoglyphs deviate from explicit chance-placement models of proximity to mapped route proxies under different definitions of possible placement space.

Geoglyphs, large-scale anthropogenic markings and constructions on ground surfaces, occur across diverse environments and cultural settings [2–4]. Research has been especially intensive in arid regions where surface visibility and preservation are high, including the Nasca-Palpa area of southern Peru [5–9]. Comparable large ground figures and landscape markings have been documented in the Atacama Desert, where formal variation and caravan-route associations have been evaluated systematically [2], and in the basalt and desert landscapes of Arabia, where satellite reconnaissance has revealed extensive distributions of ground constructions [10]. In more humid contexts, geometric ditched earthworks often discussed as “geoglyphs” are now well documented in southwestern Amazonia, with chronologies and land-use implications constrained through integrated paleoecological and radiocarbon programs [3,4].

Methodological progress in geoglyph research has been closely tied to remote sensing and near-surface imaging workflows that improve detection, mapping precision, and landscape-scale spatial analysis [11–14]. RPAS/UAV photogrammetry enables high-resolution orthomosaics and surface models suitable for documenting low-relief features and subtle ground-clearance traces [15–17]. These approaches are increasingly relevant to Peruvian case studies, including work on the Andean central coast that explicitly combines field survey with digital documentation [1,18].

A persistent limitation, however, is chronological control. Direct dating of geoglyph construction and use histories is often difficult because many features lack stratified deposits or datable construction fills; where feasible, luminescence methods (e.g., OSL) and context-linked radiocarbon programs can constrain episodes of construction and reuse [5]. Consequently, studies of ground-clearance geoglyphs frequently depend on relative chronology derived from surface-associated materials, nearby occupation sequences, and cross-feature associations, with interpretation necessarily conditioned by the inferential distance between dated contexts and the geoglyphs themselves. This limitation bears directly on spatial interpretations that relate geoglyph placement to circulation features: route geometries are typically mapped as visible linear features in the contemporary landscape, whereas their antiquity and use histories are often less securely constrained than the geoglyphs under study.

Within Peru, geoglyphs have been documented in multiple coastal valleys, yet scholarly attention remains markedly uneven. The Nasca-Palpa corpus continues to dominate the international literature (Lambers 2004, 2006; Masini et al. 2017; Sakai et al. 2023; Sakai et al. 2014), whereas the Chillón River Valley-one of the principal drainages traversing metropolitan Lima-has received comparatively limited geoglyph-specific study (Sanchez-Borjas 2024; Sanchez-Borjas et al. 2024). Relevant comparative research from other Andean regions is also available and provides an important broader context for interpretation, including work in the Sihuas Valley, northern Chile, Chincha, and recent synthetic treatment of Andean geoglyphs that emphasizes their roles not only as route markers but also as movement-organized spaces of congregation, performance, and periodic aggregation (Bikoulis et al. 2016; Bikoulis et al. 2018; Briones-M 2006; Clarkson and Briones Morales 2014; Stanish et al. 2014; Stanish et al. 2026). In the Chillón Valley, early synthetic work described linear ground-clearance features and proposed functional interpretations for cleared spaces and associated accumulations (Silva 1998; Silva 1996). Subsequent documentation at Torreblanca emphasized the placement of geoglyphs near ephemeral channels, consistent with the view that quebrada geomorphology and hydrology helped structure their location (Rodríguez 1999a, 1999b). Later research argued that several Chillón complexes occupy quebradas that function as water-collecting catchments and identified directional patterning toward the Chillón-Rímac watershed divide (Echevarría López 2015).

Building on this background, the present study documents geoglyphs in two quebradas of the middle Chillón Valley (Huarabí and Pichausa)-through systematic pedestrian survey integrated with RPAS-based recording. Chronological assessment is intentionally conservative and anchored to ceramic associations recorded in geoglyph-adjacent surface contexts; Formative-period ceramics are documented only in Huarabí, and any chronological extension beyond Huarabí is treated as provisional. The study’s second objective is inferential: to evaluate whether the documented geoglyph-to-route distances deviate from explicit Monte Carlo null models using alternative operational definitions of placement space, and to assess how sensitive those results are to the availability assumptions used in the analysis.

Accordingly, we ask:

1. Do observed geoglyph-to-route distance distributions deviate from expectations under chance placement within surveyed availability windows?
2. Is any deviation confined to corridor-restricted availability (buffer = 0 m), or does it persist under buffer-expanded windows that relax the availability definition (100–1000 m)?

Formative ceramic findspots are included only for Huarabí (n = 6) as additional unweighted point observations; no comparable ceramic dataset is available for Pichausa.

Any deviation from the null model is interpreted cautiously. Such a result identifies a difference between observed and simulated distance distributions under specified assumptions; it does not, by itself, identify intentional placement, contemporaneity with routes, or a specific behavioral mechanism.

### The formative period

Formative developments on Peru’s Andean Central Coast (Lima region) are best treated as a prolonged and internally heterogeneous transformation of Late Archaic monumental landscapes into ceramic-bearing communities, rather than as a single uniform “horizon” [19]. A Bayesian reassessment drawing on 190 radiocarbon determinations from 13 central-coast Formative sites proposes that early ceramics may be present by ~2000 cal BC at the nearby Formative site of Ancon, while several major coastal monuments are broadly coeval with early ceramic-bearing contexts at sites including Ancón, La Florida, and Garagay, within approximately 2200−1600 cal BC [19]. Under this model, ceramic adoption and “Formative” lifeways do not correspond to a single, region-wide package of material traits; instead, ceremonial centers and their associated practices may have persisted through, and overlapped with, the initial emergence of ceramics rather than being displaced by it [20].

A second trajectory emphasized for the central coast is the persistence and florescence of the U-shaped architectural tradition. U-shaped buildings are a regionally diagnostic architectural grammar with modeled activity peaking between ~1500 and 1000 cal BC and declining between ~1000 and 500 cal BC [19]. This coastal florescence is modeled as substantially overlapping with the highly influential Formative site of Chavín de Huántar, broadly contemporary with the U-shaped tradition between ~1100 and 550 cal BC, and the study highlights the appearance of Chavín-related imagery on U-shaped building facades as coincident with Chavín’s onset. Interpreted conservatively, the data indicate a chronological overlap: coastal ceremonial traditions were active during the same centuries in which Chavín became prominent, rather than being simply antecedent to highland interaction [19,21–24].

Within the Chillón Valley, eight U-shaped buildings have been identified [19,25–27], five in the lower valley and three in the middle valley. Huarabí and Pichausa lie between the middle-valley U-shaped buildings of Chocas and Pucará (27.5 km and 47.5 km apart, respectively) and correspond to the most inland expression of this architectural tradition within the valley. The two localities are separated by approximately 20 km and occupy a sector where route structure, quebrada topography, and access between valley segments may have influenced both movement and the placement of large-scale ground constructions.

The geoglyphs documented in the Huarabí and Pichausa quebradas are in proximity to 24 Formative sites (Fig 4). This spatial context justifies evaluating whether the documented geoglyphs occupy settings near mapped route proxies [28] but it does not establish that the geoglyphs were constructed during the Formative period or that they were functionally connected to specific sites. The irregular morphology shared by the Huarabí and Pichausa geoglyphs is therefore treated as a comparative observation rather than as an independent chronological marker.

## Methods

Fieldwork was conducted under Ministry of Culture’s Director’s Resolution No. 00276–2021-DCIA/MC, issued by the Dirección de Calificación de Intervenciones Arqueológicas on February 25, 2021. Both authors are members of the College of Peruvian Archaeologists (COARPE) and are registered in the Peruvian Ministry of Culture’s Register of National Archaeologists (RNA).

### Study area and spatial datasets

This study uses three main spatial datasets for each quebrada: geoglyph linework digitized from survey and RPAS documentation, mapped roads and paths, and polygons marking the documented area of survey coverage. These survey polygons define the observation universe used in the analyses. All layers were projected to UTM Zone 18S (EPSG:32718), and all distances are reported in meters [29].

The mapped roads and paths are treated as visible movement features in the present landscape. They are useful reference layers for testing spatial proximity, but they do not represent a complete inventory of ancient movement and they are not independently dated. For Pichausa, the analysis uses the mapped main-road subset rather than the full set of visible pathways. Because the route datasets differ between quebradas, all inferential tests are carried out separately.

RPAS-derived orthomosaics and related spatial products were used for documentation, digitization, and contextual mapping. Formal spatial-accuracy metadata for these products, including ground sampling distance (GSD), georeferencing RMSE, and ground-control-point error statistics, are not available for the present dataset. Positional uncertainty therefore cannot be quantified directly from the archived workflow. The spatial analyses should accordingly be read as broad-scale comparisons of geoglyph–route relationships within the surveyed area, not as high-precision tests of exact locational accuracy. Because some reported contrasts occur at relatively short distance scales, positional uncertainty may be non-trivial relative to the smaller observed differences.

### Field survey and recording

During a research project conducted in 2021–2022, systematic full-coverage pedestrian survey and drone-based mapping were undertaken in 31 zones with archaeological evidence (Sanchez-Borjas 2024; Sanchez-Borjas et al. 2024). The survey strategy was regional in scope and not restricted to route corridors: it included areas containing modern circulation features, areas with presumed earlier routes, and sectors away from both. This design is important for the present analysis because the recorded geoglyphs derive from a documented survey universe that was not confined to locations adjacent to mapped routes.

Geoglyphs were recorded in 19 zones, 13 on the north margin and 6 on the south margin. Surface ceramics were collected from geoglyph-adjacent contexts for typological assessment. Of these 19 zones, only Huarabí and Pichausa contain geoglyphs with irregular forms (Sanchez-Borjas 2024). Formative ceramics were identified only near the Huarabí geoglyphs; ceramics recorded in association with geoglyphs elsewhere in the project area belong to later chronological periods.

Because surveyed polygons are used to operationalize spatial availability, the spatial analyses evaluate route proximity conditional on the documented survey universe. Quantitative measures of within-zone survey effort (e.g., GPS track density, transect spacing, or time on ground) are not available. Consequently, detection probability may still have varied within surveyed polygons, for example because of differential accessibility or surface visibility, which could have increased the likelihood of recording both geoglyph traces and surface artifacts in some parts of the landscape, including near mapped routes. Null-model results are therefore interpreted as departures from chance expectations given documented coverage and any unmeasured within-polygon effort structure, rather than as definitive evidence that circulation independently structured geoglyph placement.

### Ceramic analysis and relative chronological assessment

Six diagnostic Formative ceramic records were documented in Huarabí surface contexts, each georeferenced and treated here as a distinct findspot for spatial analysis (Fig 5). The sample is dominated by rim fragments (5/6) and includes one decorated body sherd (1/6). Where vessel form is specified, neckless jars predominate (4/6), with one bowl (1/6). Rims are consistently described as rounded lips and continuous profiles. Wall thickness ranges from 0.70–0.90 cm (mean = 0.82 cm; n = 6). Rim diameter is reported for five records (18–27 cm; mean = 22.4 cm; n = 5). Surface treatment includes burnishing (3/6), smoothing (2/6), and polishing (1/6), with interiors predominantly smoothed; the single bowl is burnished internally. Colors fall mainly in red to reddish-brown Munsell hues (mostly 2.5YR; one 5YR 6/6). Decoration is recorded in a single specimen and is described as punctuated and incised; paint is not reported. Ceramics show resemblance to the Ancón Formative sequence, especially phases Chavinoid V and VI [30], which are contemporary with the spread of janabarroid (Chavín) ceramics in the Central Andes between 800–550 BCE [19,21–24]. However, because all sherds derive from surface contexts and the sample is small, this resemblance is treated as a coarse typological attribution rather than a basis for fine-grained intra-Formative phasing. Full catalogue attributes are provided in S4 Table.

### Inclusion of ceramic findspots

Six spatially distinct Formative ceramic findspots documented in Huarabí were incorporated as additional point observations (n = 6). Each findspot was treated as an unweighted event (one observation per location) to minimize bias arising from fragment counts, collection intensity, and surface visibility. A single nearest-route distance was calculated for each findspot using the same mapped route layer applied to the geoglyph measurements. No equivalent Formative ceramic dataset was available for Pichausa. Pichausa is therefore not assigned a secure Formative chronology in the present analysis; its irregular morphology is treated only as a comparative observation because similar forms were documented at Huarabí and were not recorded among the other geoglyphs in the surveyed area.

### Definition of analytical units

The analytical unit is the geoglyph rather than individual digitized segments. Geoglyph linework can be fragmented by preservation and recording practices; treating segments as independent observations would inflate sample size and violate independence assumptions (Banning 2002).

- Huarabí: 23 recorded segments were dissolved by geoglyph code (LAYER) into two geoglyph groups included in the analysis.
- Pichausa: two irregular geoglyph groups were retained as separate analytical units.

The analyzed geoglyph sample therefore comprises four geoglyph groups in total (two per quebrada), plus six Huarabí Formative ceramic findspots.The Formative geoglyph sample therefore comprises four geoglyph groups in total (two per quebrada), plus six Huarabí ceramic findspots.

### Mapped routes and analytical strategy

The mapped route layers are used here as explicit reference networks against which to compare the geoglyph locations. They are not assumed to be contemporary with geoglyph construction, nor to represent all ancient or ephemeral paths that may once have existed. Their value is more limited but still useful: they allow the observed geoglyph-route distances to be compared with distances generated under chance placement within the surveyed areas. Because the mapped route layers differ between Huarabí and Pichausa, the results are interpreted separately rather than pooled into a single valley-wide test.

### Within-geoglyph distance sampling

To characterize proximity to routes while preserving feature geometry, each geoglyph unit was represented by k measurement points sampled along its longest LINESTRING component. Measurement points are used to characterize within-feature distance distributions; they do not increase the number of independent archaeological observations. For each measurement point, distance to the nearest mapped route was computed as the minimum Euclidean distance to the route layer for the same quebrada.

### Null model: shape-preserving spatial randomization

#### Shape-preserving null model for geoglyphs.

In each Monte Carlo replicate, each geoglyph unit is represented by its longest LINESTRING component (“template”). For each template, we generate a chance placement within the availability window by applying (i) a random rotation about the template centroid (mean of vertex coordinates), and (ii) a random translation by sampling a target centroid location uniformly within the availability window polygon (sf::st_sample, type = “random”). To reduce boundary rejections in narrow corridors, centroid locations are sampled from an inset window produced by buffering the availability polygon inward by the template radius (maximum centroid-to-vertex distance) when the inset has positive area; otherwise, sampling uses the full window. A proposed placement is accepted only if the entire transformed template is contained within the availability window (sf::st_covered_by); otherwise, it is rejected and resampled, up to 5000 attempts per template per replicate. Route geometries are held fixed. These null tests evaluate whether the observed geoglyph-to-route distance distribution could arise if identical geoglyph forms were positioned without reference to routes, conditional on the specified availability definition.

#### Scenario-level test statistics, acceptance rates, and placement-attempt diagnostics are reported in S1 Table; placement-attempt diagnostics are visualized in S2 Fig. Point-randomization null model for Huarabí ceramics.

For Huarabí ceramics, in each replicate we sample points uniformly at random within the Huarabí availability window (sf::st_sample, type = “random”) and compute distances to mapped routes. This preserves the observed event count while treating ceramic locations as spatially exchangeable under the null [31].

#### ECDF grid specification (reported for interpretability).

Empirical cumulative distribution functions (ECDFs) were evaluated on a fixed distance grid spanning the observed and simulated distance ranges for each condition. To ensure reproducibility, random-number generation used fixed seeds set deterministically for each condition (quebrada × availability window × k). Scenario-level diagnostics, median-based effect sizes, and k-sensitivity results are reported in S1 Table, S2 Table, and S3 Table, respectively.

### Availability window and sensitivity analysis

Availability was defined primarily as the surveyed polygon for each quebrada (buffer = 0 m), corresponding to documented field coverage and treated as the primary inferential condition. Because surveyed boundaries may underestimate feasible placement space, a window-sensitivity sweep was conducted by buffering surveyed polygons by 100, 250, 500, and 1000 m. Buffer-expanded windows are reported as sensitivity diagnostics showing how inference changes under alternative hypothetical definitions of feasible placement space; they are not treated as independent confirmatory tests. These windows are geometric proxies for potential placement space and should not be read as terrain-based models of construction feasibility. The geomorphic feasible-window specifications used in the sensitivity analyses are shown in S1 Fig.Test statistics, effect sizes, and Monte Carlo p-values

Observed and simulated nearest-route distance distributions were compared using a non-directional discrepancy measure based on the integrated squared difference between the observed ECDF and the simulation-mean ECDF evaluated on a common distance grid. For each condition, the same discrepancy was computed for every Monte Carlo replicate. A Monte Carlo p-value was then obtained as the proportion of replicates with discrepancy at least as large as the observed value, using a standard finite-simulation correction to avoid zero p-values. To support interpretation of direction and magnitude, we also report median-based summaries: the observed median, the median of simulated medians, their difference (Δmedian), and the central 95% interval of simulated medians.

### Robustness to within-geoglyph sampling density

To reduce dependence on the chosen point density (k), analyses were repeated with k = 10, 20, and 40 under the primary window (buffer = 0 m). This evaluates whether inference is driven by within-feature discretization rather than by feature placement relative to routes. Results of this k-sensitivity analysis are reported in S3 Table; the corresponding ECDF envelopes are shown in S4 Fig. Statistical considerations and limitations

The analyses quantify spatial association under explicitly stated assumptions; they do not establish deterministic behavioral mechanisms.

1) Small empirical sample. The Formative dataset comprises four geoglyph groups (plus six ceramic findspots in Huarabí). Inference is therefore constrained and should be interpreted as evidence about these recorded contexts rather than as a valley-wide generalization.
2) Model-conditional feasibility. The null model assumes uniform placement feasibility within each availability window. This is a simplifying assumption rather than a terrain-based model of construction suitability. Because slope, substrate, surface stability, microtopographic visibility, erosion, and geomorphic constraints are not modeled directly, some observed deviations may reflect misspecification of the counterfactual placement space rather than cultural siting preferences. This limitation restricts the analysis to exploratory, model-dependent inference.
3) Monte Carlo uncertainty and multiplicity. Monte Carlo p-values were estimated from 999 simulations using a standard finite-simulation correction. With 999 simulations, sampling uncertainty is non-trivial near conventional thresholds: for an estimated p-value near 0.05, the Monte Carlo standard error is about 0.007, implying a rough 95% Monte Carlo uncertainty band of approximately ±0.014. Because multiple buffers (and k values) are evaluated as sensitivity diagnostics, near-threshold outcomes are interpreted cautiously; emphasis is placed on robustness patterns (direction and magnitude of Δmedian and consistency across k) rather than dichotomous thresholding.
4) Availability sensitivity. Buffering changes the null distance distribution and can change both the direction and strength of deviation; window sweeps are therefore interpreted as sensitivity diagnostics that bound inference to explicit availability assumptions rather than as independent confirmatory tests.
5) Route proxies. Results pertain to the mapped route datasets used here (including the Pichausa main-road subset). Undocumented or ephemeral pathways may have influenced placement, and alternative route representations could change effect magnitudes and p-values.
6) Survey-effort uncertainty. Because quantitative effort surfaces are unavailable, undiagnosed within-polygon effort gradients could contribute to apparent route proximity. Results are interpreted conditional on documented coverage and potential within-polygon heterogeneity in detectability.
7) Spatial accuracy of the RPAS-derived mapping products is an additional limitation. Because formal metadata documenting image resolution, georeferencing error, and ground-control performance are unavailable, the positional uncertainty of mapped geoglyph and route geometries cannot be quantified directly. This does not invalidate the descriptive and exploratory spatial analyses presented here, but it does require caution in interpreting effects that operate at relatively short distance scales. The results are therefore treated as evidence of broad spatial discrepancy under stated assumptions rather than as precise locational estimates of behavioral attraction to routes.

The availability windows used here are geometric approximations of potential placement space rather than independently modeled construction-feasibility surfaces. They do not incorporate bare-earth DTM-derived slope, microtopography, substrate, surface clast density, visibility of cleared lines, erosion history, or geomorphic stability. Consequently, the null model evaluates whether observed geoglyph and ceramic distances differ from random placements within the specified spatial windows, but it cannot determine whether those deviations arise from cultural siting preferences, geomorphic constraints, or misspecification of the counterfactual placement space.

Analytical scripts are provided as supporting code: S1 Code constructs the screened geomorphic feasible windows, S2 Code constructs the strict geomorphic feasible windows, S3 Code runs the Monte Carlo analyses and exports the scenario-level, effect-size, and k-sensitivity outputs, and S4 Code regenerates the supplementary figures from S3 Code outputsResults

### Route proximity under corridor-restricted availability (buffer = 0 m)

Under the survey-polygon window (buffer = 0 m), the observed geoglyphs and ceramic findspots occupy settings that are accessible within quebrada topography (Table 1). In Huarabí, both geoglyphs and the six Formative ceramic findspots lie mostly within about 200 m of mapped routes. In Pichausa, both geoglyphs also lie within tens of meters of mapped main roads. These values describe the observed pattern only; they do not by themselves show whether the pattern differs from chance.

**Table 1:** Observed distances to the nearest mapped route (buffer = 0 m).

Site	Feature type	Group/ unit	n (distance measurements)	Median distance (m)	Range (m)
Huarabí	Ceramic	HUARABI_CERAMICS	6	70.8	34.0-178.4
Huarabí	Geoglyph	HUARABI_G_T1	20	90.7	40.0-143.4
Huarabí	Geoglyph	HUARABI_G_T4	20	136.2	45.4-183.3
Pichausa	Geoglyph	PICHAUSA_01	20	73.0	61.3-85.5
Pichausa	Geoglyph	PICHAUSA_02	20	54.6	35.6-70.7

Observed Euclidean distances (m) from (i) within-geoglyph measurement points (k = 20 points sampled along the longest linestring component of each geoglyph unit) and (ii) unweighted Huarabí Formative ceramic findspots (n = 6; one observation per location) to the nearest mapped route segment. Values summarize the observed data only (median and range). Measurement points are used to characterize within-feature distance distributions and do not constitute independent archaeological observations.

When the observed distances are compared with the null model, Huarabí departs from the tested null under the buffer = 0 m window, whereas Pichausa does not (Table 2). In Huarabí, the observed median is greater than the simulated median, so the result is not consistent with a simple attraction to the nearest route. The safest reading is narrower: under the surveyed window and relative to the mapped route layer used here, the Huarabí configuration is less well reproduced by the tested null model than the Pichausa configuration.

**Table 2:** Monte Carlo test results and effect sizes under corridor-restricted availability (buffer = 0 m; primary analysis).

Dataset	Buffer (m)	n	ECDF discrepancy (T_obs)	Monte Carlo p	Median_obs (m)	Median_sim (m)	Δ median (m)	Simulated median 95% interval (m)
Huarabí (geoglyphs + ceramics)	0	46	17.240	0.021	100.8	43.3	57.5	11.0-111.3
Pichausa (geoglyphs)	0	40	6.643	0.380	65.4	48.5	16.9	11.4-115.5

Comparison of observed nearest-route distance distributions to a shape-preserving Monte Carlo null model (999 simulations). In each simulation, each geoglyph template is randomly rotated and translated within the surveyed polygon (buffer = 0 m) while holding route geometries fixed; Huarabí ceramics are modeled with a point-randomization null that samples n = 6 points uniformly within the same window. Results are reported separately for Huarabí (geoglyph distances + ceramics) and Pichausa (geoglyph distances). Monte Carlo p-values quantify evidence for deviation from the null under stated assumptions using a global ECDF discrepancy statistic (Cramér-von Mises-type). Effect size is summarized as Δmedian = median(observed distances) − median(simulated medians), with the 2.5th-97.5th percentile interval of simulated medians.

Table 2 presents the primary inferential result under the survey-polygon window (buffer = 0 m).

### Sensitivity to availability definition: buffer-defined and geomorphically screened windows

The buffer sweep shows that the results depend strongly on how possible placement space is defined (S1 Table, S2 Table, and S3 Fig).At 100–250 m, both quebradas remain consistent with the null. At 500 m, neither departs from it. At 1000 m, Huarabí shows a larger deviation from the tested null model, whereas Pichausa yields a near-threshold result that should be treated cautiously. Median differences also reverse sign as the window expands. This indicates that broadening the possible placement area shifts the simulated distance distribution upward and can make the observed features appear relatively closer to mapped routes by comparison. For that reason, the buffer sweep should be read as a sensitivity test rather than as cumulative support for a single interpretation.

Null scenario-level statistics and median-based effect sizes for the geomorphic specifications are reported in S1 Table, and S2 Table; the feasible-window specifications are shown in S1 Fig.

## Discussion

This study documents previously unpublished geoglyph contexts in two quebradas of the middle Chillón drainage, Huarabí and Pichausa, and evaluates their relationship to mapped circulation features using explicit counterfactual (“chance placement”) models. Descriptively, geoglyph sampling points and Huarabí Formative ceramic findspots lie at distances of tens to low hundreds of meters from mapped routes. This pattern is compatible with placement in accessible quebrada settings where movement is constrained by relief and corridor geometry, but it does not by itself ‌‌demonstrate deliberate association with specific routes.

### Movement landscapes, availability, and interpretive limits

All inferences remain conditional on the mapped route proxies and do not establish contemporaneity between routes and geoglyph construction. Under the primary survey-polygon window (buffer = 0 m), Huarabí departs from the tested null whereas Pichausa does not. The direction of the Huarabí result is important: the observed median exceeds the simulated median, so the pattern is not consistent with a simple rule of minimizing distance to routes.

The narrowest defensible interpretation is therefore that, within the documented survey universe and relative to the mapped route layer used here, the Huarabí configuration is less well reproduced by the tested null model than the Pichausa configuration.

The buffer sweep shows that this inference depends strongly on how possible placement space is defined. At 100–250 m, both quebradas remain consistent with the null; at 500 m, neither departs from it; at 1000 m, Huarabí shows a larger deviation from the tested null model, whereas Pichausa yields a near-threshold result that should be treated cautiously. The sign reversal in Δmedian across broader windows indicates that expanding the opportunity space shifts the simulated distance distribution upward and can make the observed features appear relatively closer to mapped routes by comparison. For that reason, the buffer sweep is best read as a sensitivity test rather than as cumulative confirmation of a single route-related process.

The geomorphic analyses point in the same general direction. Under manually screened feasible windows, Huarabí still departs from the null and Pichausa still does not. These scenarios reduce obviously implausible placement space, but they remain exploratory because they are based on manual screening rather than on a bare-earth DTM and therefore do not model slope, curvature, roughness, substrate stability, or other terrain variables directly. More broadly, the ECDF statistic used here is non-directional: a departure from the null indicates a difference between observed and simulated distance distributions under the stated assumptions, not a single behavioral mechanism. The current results therefore support only a model-dependent spatial discrepancy in Huarabí, not a demonstrated case of intentional route association or broader spatial organization. Alternative explanations, including geomorphic channeling, visibility, threshold marking, recurrent movement, or survey-process effects, cannot be distinguished with the present dataset.

### Chronology and cultural placement in the formative landscape

Chronological attribution remains one of the principal limits of this study. Huarabí provides the only currently documented contextual indication of nearby Formative-period activity, based on diagnostic surface ceramics consistent with Ancón-related Formative traditions. Even there, however, the evidence must be bounded carefully. Because the material derives from surface contexts, its depositional relationship to the linework cannot be established securely: the fragments may predate, postdate, or be broadly contemporary with geoglyph construction. The small size of the sample further limits chronological resolution. The Huarabí ceramics are therefore better interpreted as evidence of Formative-period activity in the vicinity of one geoglyph locality than as direct proof of geoglyph construction age. In Pichausa, where no comparable diagnostic material or dated adjacent deposits were recorded, chronological attribution remains provisional.

This chronological asymmetry makes Huarabí more informative than Pichausa only in a restricted sense. Its value lies in showing that Formative-period activity occurred near one documented geoglyph locality, not in securely dating the geoglyphs themselves. The surrounding distribution of Formative sites and U-shaped ceremonial architecture provides a relevant archaeological context for future interpretation, but the present evidence does not demonstrate that the geoglyphs marked formal routes, structured ritual movement, or formed part of a single cultural mechanism. At present, Huarabí is best understood as a geoglyph locality with nearby Formative-period surface activity, embedded within a broader middle-valley archaeological landscape.

### Bounding inference given survey effort and route proxies

Two issues remain especially important for interpretation. First, the mapped route layers are proxies for visible circulation structure in the contemporary landscape. They are analytically useful because they provide an explicit reference network against which chance-placement expectations can be evaluated, but they are not independently dated and they do not capture the full set of ancient or ephemeral pathways that may once have existed. The results therefore pertain strictly to relationships between the recorded geoglyph contexts and the mapped route datasets used here.

Second, the null remains conditional on imperfect knowledge of feasibility and detectability. Although the survey design was regional rather than route-focused, quantitative effort surfaces are unavailable, so subtle within-polygon gradients in accessibility or visibility could still have affected where geoglyphs and artifacts were recorded. Likewise, even after manual geomorphic screening, the analysis does not yet include terrain-derived constraints independent of the mapped routes themselves. This distinction is crucial: general movement channeling imposed by quebrada topography is not the same thing as deliberate association with a particular route network. Without more explicit terrain and visibility modeling, apparent route proximity may partly reflect geomorphological channeling and survey process rather than intentional siting relative to circulation corridors.

### Implications for interpretation

Taken together, the results justify a narrow but worthwhile conclusion. Huarabí is less well reproduced by the tested null models than Pichausa, and that difference remains under conservative geomorphic screening. Pichausa, by contrast, remains consistent with the null across the baseline and geomorphic scenarios. The contrast does not guarantee a strong claim that one quebrada hosted deliberately route-oriented geoglyph construction while the other did not. Rather, it indicates that the Huarabí configuration is less easily reproduced by the null models tested here, whereas the Pichausa configuration is not.

Archaeologically, the Huarabí result is best treated as a basis for further testing rather than as a substantive explanation. In a middle-valley sector that includes U-shaped ceremonial architecture and multiple Formative sites, the co-occurrence of irregular geoglyphs and nearby Formative-period ceramic activity raises questions about movement, encounter, and landscape use within quebrada corridors [1,28]. However, those questions remain hypotheses. The current evidence is sufficient to define a reproducible problem for future research, not to resolve whether the geoglyphs were intentionally associated with routes or with specific ritual practices.

### Priorities for future testing

Several steps would materially improve identifiability while preserving the counterfactual logic of the present analysis.

1. Model feasibility more explicitly. A next-generation analysis should incorporate slope, substrate, and geomorphic suitability so that null placements are sampled only from terrain that was physically plausible for geoglyph construction.
2. Evaluate visibility and encounter structure. Intervisibility, approach-route visibility, and vantage-based modeling would help distinguish corridor marking from other forms of placement.
3. Secure independent chronology. Deposits physically adjacent to linework, and where possible selected route segments, should be targeted for dating and contextual association.
4. Standardize route representations. Comparative tests using harmonized route definitions across quebradas would reduce interpretive asymmetry.
5. Integrate settlement history more directly. The relationship between these geoglyph locales, nearby Formative sites, and the middle-valley U-shaped building sector should be developed at a multiscalar level.

Together, these steps would permit more discriminating tests of whether the observed proximity patterns reflect cultural siting practices, geomorphic constraints, visibility conditions, survey-process effects, or some combination of these factors.

## Conclusions

Within the 2021–2022 survey universe, Huarabí is the only quebrada in which diagnostic Formative ceramics were recorded near geoglyph linework. This evidence indicates nearby Formative-period activity, but it does not directly date geoglyph construction or establish cultural attribution. Pichausa remains chronologically less certain.

In both quebradas, the geoglyphs lie in accessible corridor settings and at short distances from the mapped route layers used here. Under the primary survey-polygon window, however, only Huarabí deviates from the tested null model. That difference remains visible under the geomorphic sensitivity tests, but the broader buffer sweep shows that interpretation depends on how possible placement space is defined. The results should therefore be read as exploratory, model-dependent evidence of difference from tested null models, not as proof of a single cultural mechanism or deliberate attraction to routes.

At present, Huarabí is best understood not as a securely dated Formative geoglyph complex, but as a geoglyph locality with nearby Formative-period surface activity. More robust inference will require terrain-based feasibility modeling, visibility analysis, and independent chronology from deposits adjacent to geoglyph linework and, where possible, from selected route segments.

## Supporting information

S1 Table Monte Carlo test statistics and placement diagnostics across buffer-defined and geomorphic availability scenarios. (DOCX)

S2 Table Median-based effect sizes across buffer-defined and geomorphic availability scenarios. (DOCX)

S3 Table Sensitivity of Monte Carlo results to within-geoglyph sampling density under the screened geomorphic scenario. (DOCX)

S4 Table Catalogue of diagnostic Formative ceramics from Huarabí surface contexts. (DOCX)

S1 Fig Geomorphic feasible-window specifications used in sensitivity analyses. (PNG)

S2 Fig Monte Carlo placement diagnostics by availability scenario. (PNG)

S3 Fig Observed versus simulated median nearest-route distances by availability scenario. (PNG)

S4 Fig k-sensitivity ECDF envelopes under the screened geomorphic scenario. (PNG)

S1 Code Builds the screened geomorphic exclusion masks for Huarabí and Pichausa from georeferenced RGB terrain imagery using an interactive mapedit workflow in R. The script reads the survey polygons, route layers, geoglyph layers, and Huarabí ceramic points, allows the user to digitize exclusion polygons manually, converts those exclusions into feasible windows, checks whether observed geoglyphs and ceramics remain inside the retained window, and writes GeoPackage and CSV outputs summarizing exclusion area, feasible area, and retained proportion for each site.
(R)

S2 Code This is the same exclusion-mask builder as the screened version but configured for the strict/ second-pass geomorphic scenario. In practice, it generates the alternative stricter feasible windows used later in sensitivity analysis. The script includes a more robust package installer, removes the unused lwgeom dependency, and fails clearly if the interactive mapping packages are unavailable. Its outputs are the exclusion polygons, feasible-window GeoPackage, and a mask-summary.
(R)

S3 Code Runs the main geomorphically screened shape-preserving Monte Carlo analysis for the Formative geoglyph dataset and the Huarabí ceramic points. It reads the original survey, route, geoglyph, and ceramic layers; imports the screened and strict feasible-window outputs from Script R01; rebuilds feasible windows automatically if the GeoPackages are missing or empty; samples points along geoglyph templates; computes nearest-route distances; performs 999 Monte Carlo simulations across both buffer-defined and geomorphic scenarios; calculates ECDF-based discrepancy statistics, Monte Carlo p-values, median-based effect sizes, and k-sensitivity results; and writes the scenario/effect/k-sensitivity CSV outputs used in the manuscript and SI. It also exports the SI figures for geomorphic windows, placement diagnostics, observed-versus-simulated medians, and ECDF k-sensitivity.
(R)

S4 Code Rebuilds the supplementary figures only, using the CSV outputs generated by R02 rather than rerunning the full Monte Carlo analysis. Specifically, it reads the scenario-level statistics, median-effect summaries, and optional ECDF k-sensitivity file, then regenerates the TIFF versions of the SI figures for placement diagnostics, observed-versus-simulated.
(R)
