# Supplementary material for: Geoglyphs and formative-period activity in the middle Chillón Valley, Peru: Ceramic association and null-model tests of route proximity
Source: PLoS One. 2026 Jun 8;21(6):e0350855. doi: 10.1371/journal.pone.0350855 (PMC13245780; doi:10.1371/journal.pone.0350855)
Supplement: S1 Table — (DOCX) [file pone.0350855.s001.docx]

Table S1. Monte Carlo test statistics and placement diagnostics across buffer-defined and geomorphic availability scenarios

| **Scenario** | **Scenario type** | **Huarabí feasible area (ha)** | **Huarabí T_obs** | **Huarabí p** | **Huarabí acceptance rate** | **Pichausa feasible area (ha)** | **Pichausa T_obs** | **Pichausa-p** | **Pichausa acceptance rate** |
| --- | --- | --- | --- | --- | --- | --- | --- | --- | --- |
| Survey polygon (0 m) | Buffer | 46.79 | 17.240 | 0.021 | 0.179 | 27.95 | 6.643 | 0.380 | 1.000 |
| Survey +100 m | Buffer | 89.14 | 7.672 | 0.396 | 0.391 | 52.96 | 6.987 | 0.471 | 1.000 |
| Survey +250 m | Buffer | 164.09 | 7.802 | 0.559 | 1.000 | 102.16 | 10.692 | 0.544 | 1.000 |
| Survey +500 m | Buffer | 319.85 | 48.445 | 0.102 | 1.000 | 215.43 | 55.164 | 0.184 | 1.000 |
| Survey +1000 m | Buffer | 748.03 | 236.273 | 0.001 | 1.000 | 559.65 | 207.979 | 0.046 | 1.000 |
| Screened geomorphic window | Geomorphic | 44.30 | 16.205 | 0.022 | 0.122 | 27.69 | 6.597 | 0.433 | 1.000 |
| Alternative geomorphic mask | Geomorphic | 45.20 | 16.956 | 0.025 | 0.139 | 27.65 | 6.752 | 0.418 | 1.000 |

**Note.** T_obs is the observed ECDF discrepancy statistic. Acceptance rate is the proportion of valid shape-preserving random placements accepted under each availability specification.
