# Supplementary material for: Geoglyphs and formative-period activity in the middle Chillón Valley, Peru: Ceramic association and null-model tests of route proximity
Source: PLoS One. 2026 Jun 8;21(6):e0350855. doi: 10.1371/journal.pone.0350855 (PMC13245780; doi:10.1371/journal.pone.0350855)
Supplement: S2 Table — (DOCX) [file pone.0350855.s002.docx]

Table S2. Median-based effect sizes across buffer-defined and geomorphic availability scenarios

| **Scenario** | **Huarabí observed median (m)** | **Huarabí simulated median (m)** | **Huarabí Δmedian (m)** | **Huarabí simulated median 95% interval (m)** | **Pichausa observed median (m)** | **Pichausa simulated median (m)** | **Pichausa Δmedian (m)** | **Pichausa simulated median 95% interval (m)** |
| --- | --- | --- | --- | --- | --- | --- | --- | --- |
| Survey polygon (0 m) | 100.8 | 43.3 | 57.5 | 11.0–110.3 | 65.4 | 48.5 | 16.9 | 11.4–115.5 |
| Survey +100 m | 100.8 | 62.3 | 38.5 | 15.5–183.6 | 65.4 | 50.2 | 15.3 | 9.0–127.6 |
| Survey +250 m | 100.8 | 117.3 | -16.5 | 19.7–261.6 | 65.4 | 78.8 | -13.4 | 11.2–195.1 |
| Survey +500 m | 100.8 | 236.3 | -135.5 | 39.4–464.1 | 65.4 | 181.3 | -115.8 | 20.8–385.5 |
| Survey +1000 m | 100.8 | 561.9 | -461.1 | 131.2–909.3 | 65.4 | 435.5 | -370.1 | 53.9–799.7 |
| Screened geomorphic window | 100.8 | 45.1 | 55.7 | 10.7–111.5 | 65.4 | 48.1 | 17.3 | 12.2–117.1 |
| Alternative geomorphic mask | 100.8 | 44.6 | 56.2 | 10.9–112.8 | 65.4 | 49.1 | 16.3 | 11.0–112.5 |

Note. Δmedian = observed median − simulated median. Positive values indicate that observed median nearest-route distances exceed the simulation-based median expectation under the specified availability scenario; negative values indicate the opposite.
