# Supplementary material for: Geoglyphs and formative-period activity in the middle Chillón Valley, Peru: Ceramic association and null-model tests of route proximity
Source: PLoS One. 2026 Jun 8;21(6):e0350855. doi: 10.1371/journal.pone.0350855 (PMC13245780; doi:10.1371/journal.pone.0350855)
Supplement: S3 Table — (DOCX) [file pone.0350855.s003.docx]

Table S3. Sensitivity of Monte Carlo results to within-geoglyph sampling density under the screened geomorphic scenario

| **k points per geoglyph** | **Huarabí p** | **Huarabí Δmedian (m)** | **Huarabí simulated median 95% interval (m)** | **Pichausa p** | **Pichausa Δmedian (m)** | **Pichausa simulated median 95% interval (m)** |
| --- | --- | --- | --- | --- | --- | --- |
| 10 | 0.051 | 46.9 | 11.1–110.9 | 0.388 | 18.2 | 11.5–112.9 |
| 20 | 0.022 | 55.7 | 10.7–111.5 | 0.433 | 17.3 | 12.2–117.1 |
| 40 | 0.033 | 54.6 | 10.7–113.3 | 0.392 | 16.2 | 11.6–115.5 |

Note. Results are shown for the screened geomorphic availability scenario only. Variation in k evaluates whether inference is materially affected by within-geoglyph sampling density.
