# Supplementary material for: Geoglyphs and formative-period activity in the middle Chillón Valley, Peru: Ceramic association and null-model tests of route proximity
Source: PLoS One. 2026 Jun 8;21(6):e0350855. doi: 10.1371/journal.pone.0350855 (PMC13245780; doi:10.1371/journal.pone.0350855)
Supplement: S4 Table — (DOCX) [file pone.0350855.s004.docx]

Table S4. Catalogue of diagnostic Formative ceramics from Huarabí surface contexts.

| **Code** | **Site** | **UTM (N/E)** | **Specific type** | **Morphology** | **Wall thick. (cm)** | **Lip** | **Rim** | **Mouth diam. (cm)** | **Paste** | **Surface finish** | **Interior finish** | **Munsell** | **Decoration** |
| --- | --- | --- | --- | --- | --- | --- | --- | --- | --- | --- | --- | --- | --- |
| M-25-01 | Huarabí | 8709442 N; 293907 E | Diagnostic rim | Neckless pot | 0.8 | Rounded | Continuous | 27 | Coarse brown | Smoothed | Smoothed | 2.5YR 5/6 |  |
| M-31-01 | Huarabí | 8709266 N; 293992 E | Diagnostic rim | Neckless pot | 0.9 | Rounded | Continuous | 20 | Medium brown | Burnished | Smoothed | 2.5YR 5/6 |  |
| M-33-01 | Huarabí | 8709710 N; 293991 E | Diagnostic rim | Bowl | 0.8 | Rounded | Continuous | 25 | Medium orange | Burnished | Burnished | 5YR 6/6 |  |
| M-34-01 | Huarabí | 8709703 N; 293992 E | Diagnostic rim | Neckless pot | 0.9 | Rounded | Continuous | 18 | Coarse brown | Smoothed | Smoothed | 2.5YR 6/8 |  |
| M-35-01 | Huarabí | 8709732 N; 293891 E | Decorated body sherd |  | 0.7 |  |  |  | Fine brown | Polished | Smoothed | 2.5YR 5/8 | Punctated and incised |
| M-38-03 | Huarabí | 8709700 N; 293984 E | Diagnostic rim | Neckless pot | 0.8 | Rounded | Continuous | 22 | Medium brown | Burnished | Smoothed | 2.5YR 5/6 |  |
