# Supplementary figures and images for: Geoglyphs and formative-period activity in the middle Chillón Valley, Peru: Ceramic association and null-model tests of route proximity
Source: PLoS One. 2026 Jun 8;21(6):e0350855. doi: 10.1371/journal.pone.0350855 (PMC13245780; doi:10.1371/journal.pone.0350855)

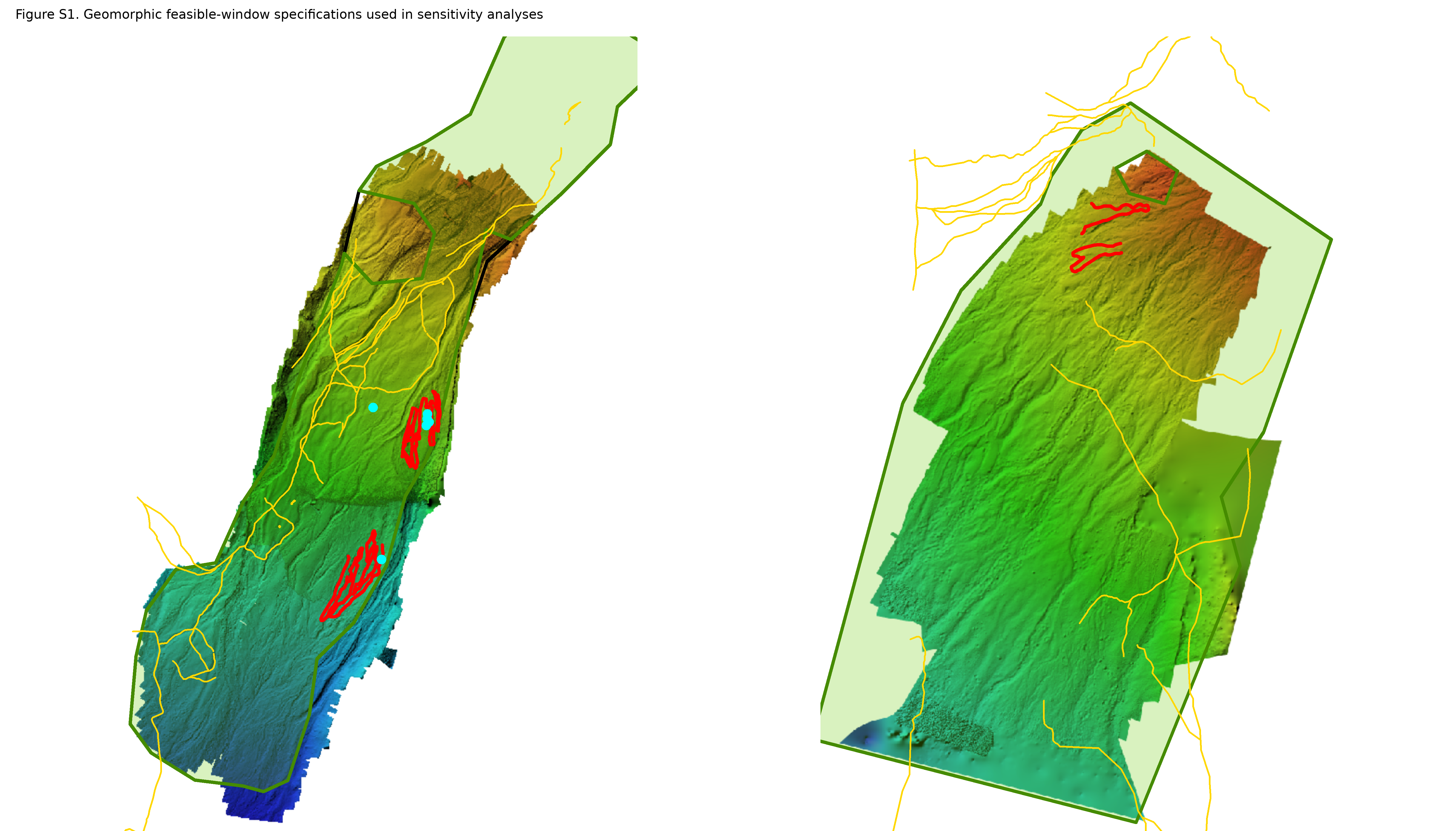

Supplement: S1 Fig — (PNG) [file pone.0350855.s005.png]

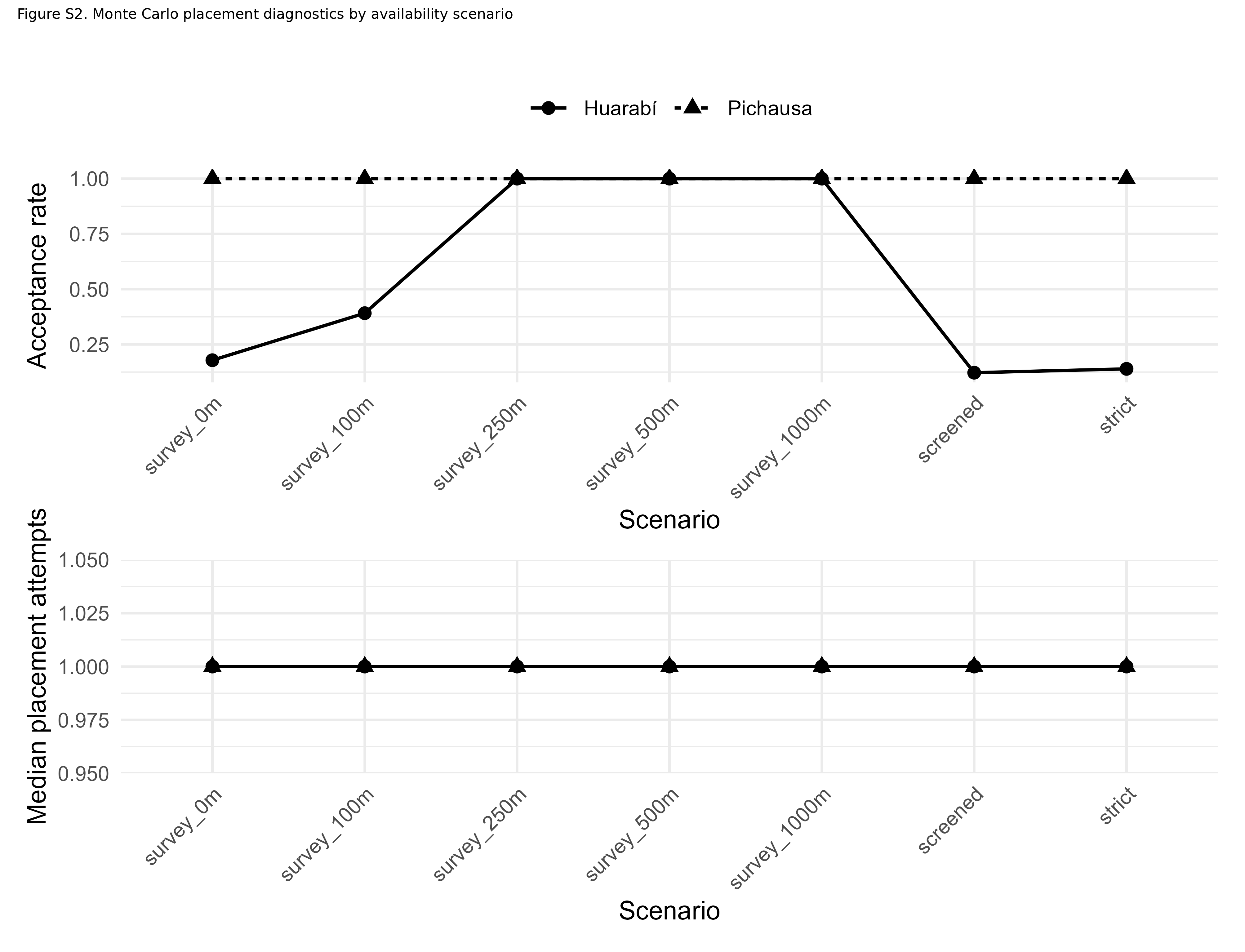

Supplement: S2 Fig — (PNG) [file pone.0350855.s006.png]

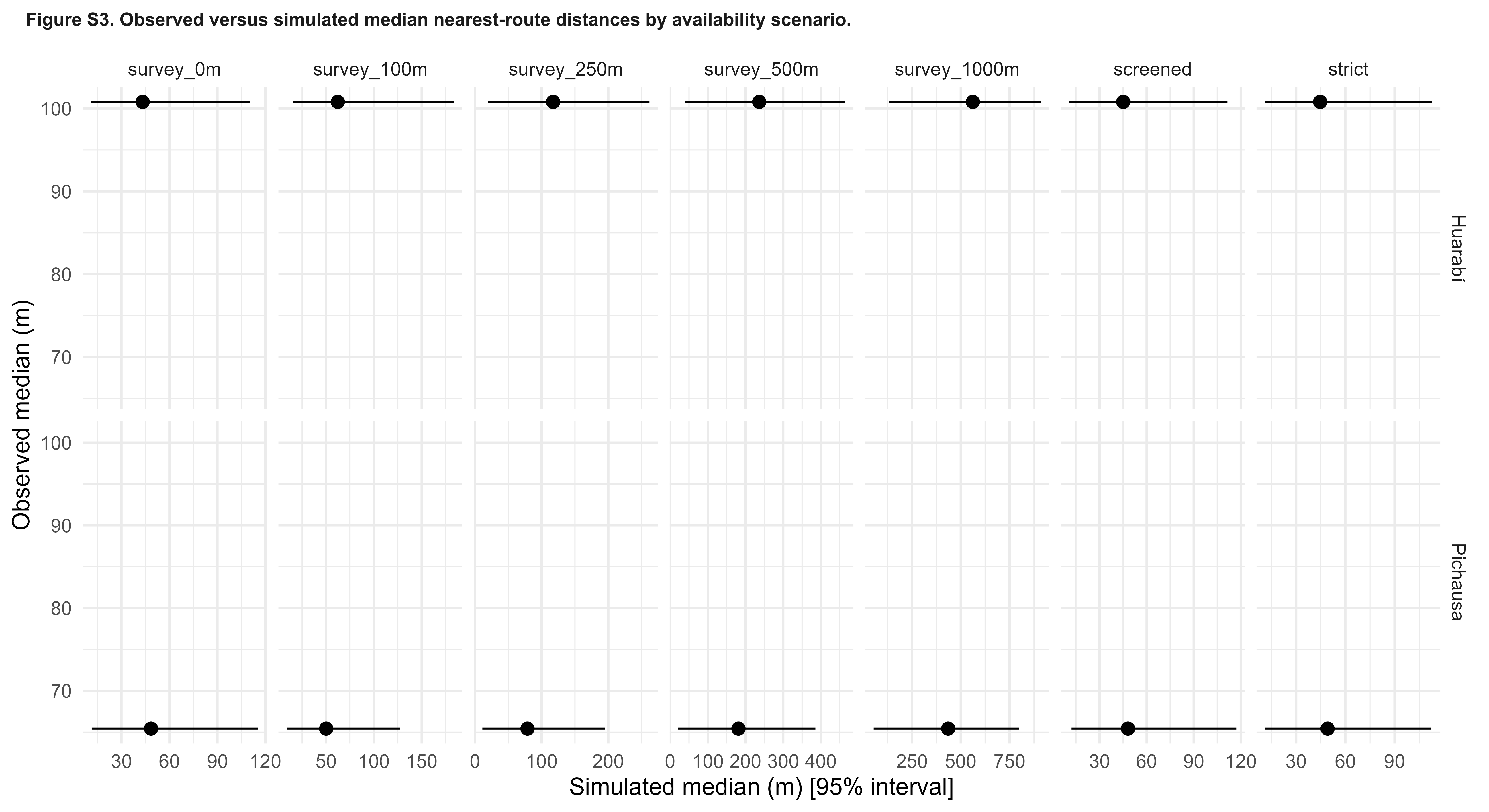

Supplement: S3 Fig — (PNG) [file pone.0350855.s007.png]

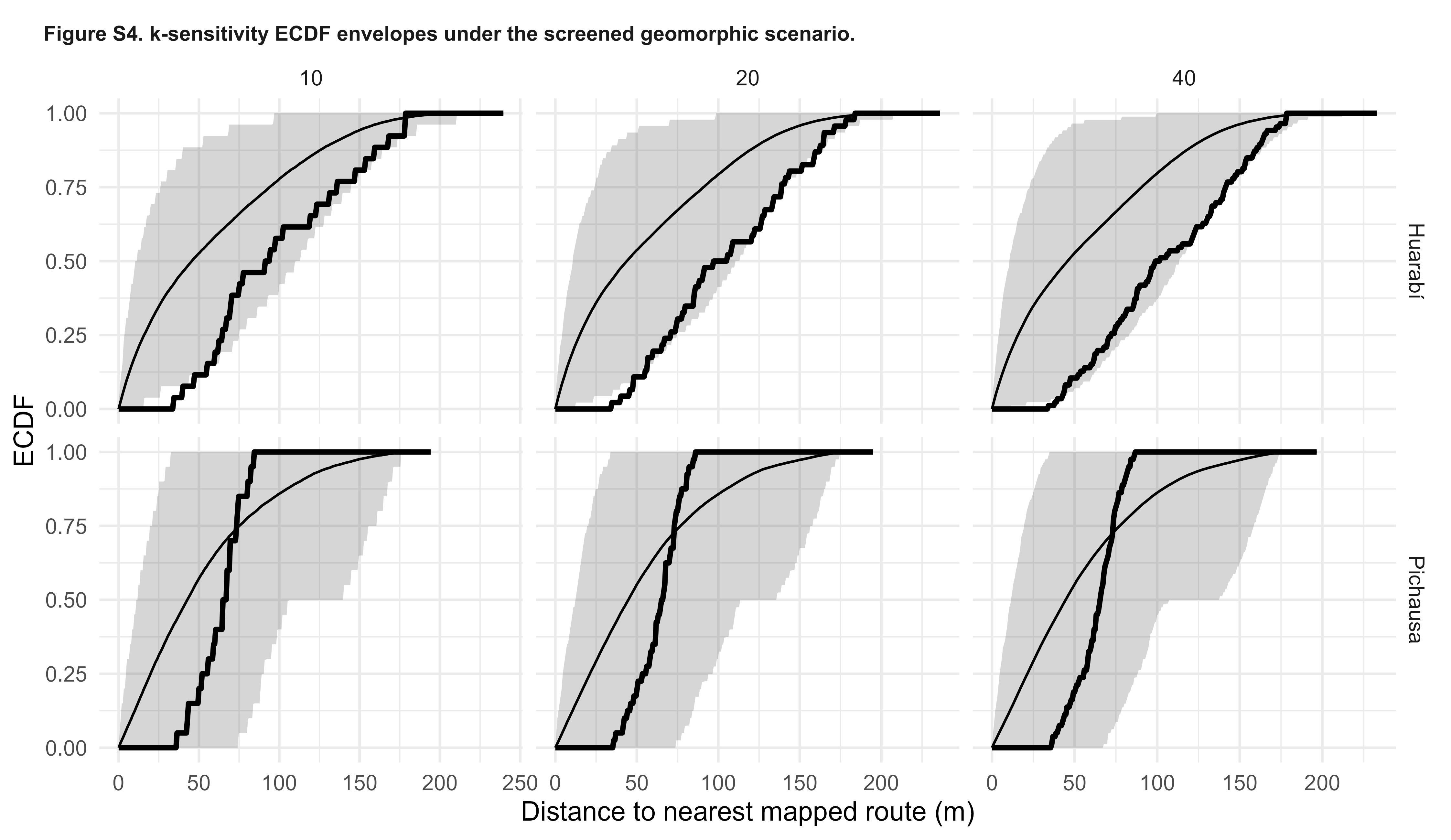

Supplement: S4 Fig — (PNG) [file pone.0350855.s008.png]
