# Supplementary material for: Geoglyphs and formative-period activity in the middle Chillón Valley, Peru: Ceramic association and null-model tests of route proximity
Source: PLoS One. 2026 Jun 8;21(6):e0350855. doi: 10.1371/journal.pone.0350855 (PMC13245780; doi:10.1371/journal.pone.0350855)
Supplement: Attachment — Submitted filename: Letter to reviewers.docx [file pone.0350855.s014.docx]

Lima, April 9^th^, 2026

We thank the Editor and the reviewers for their careful and constructive evaluations. In response, we revised the manuscript substantially. The revised version now clarifies the relationship of this article to the earlier survey overview, narrows the chronological claim, expands the archaeological framing of Huarabí and Pichausa within the middle Chillón Valley, clarifies the status of mapped routes as present-landscape proxies, states the regional rather than route-targeted character of the survey design, makes the inferential limits of the spatial analysis more explicit, adds an explicit RPAS-accuracy limitation, and includes a dedicated ethics statement with permit information. We also reorganized the supporting information so that buffer sweeps, geomorphic scenarios, and k-sensitivity analyses are presented clearly as sensitivity diagnostics rather than as primary inferential results.

**Reviewer 1**

| **Reviewer comment** | **Response** | **Change made in revised manuscript** |
| --- | --- | --- |
| The dataset is small and would be stronger if integrated into a broader archaeological context rather than treated primarily through simulation. | We agree. The revision now gives much greater weight to the broader middle-valley Formative setting. The simulation results are retained, but they are no longer treated as the only or even the main archaeological payoff of the paper. | Added a fuller Formative-period section, explicit middle-valley context, and a revised Discussion that situates Huarabí within a broader Formative archaeological landscape rather than using the statistics alone as the principal interpretive frame. |
| Why were Huarabí and Pichausa selected, given the larger survey universe? | We now explain this directly. The present article does not treat all geoglyphs recorded in the broader project. It focuses on the two quebradas that contain the irregular geoglyph forms under discussion; Huarabí is additionally important because it yielded diagnostic Formative ceramics in geoglyph-adjacent surface contexts. | Clarified in *Field survey and recording* that geoglyphs were recorded in 19 zones, but only Huarabí and Pichausa contain the irregular forms analyzed here. |
| Were the six Formative sherds the only surface finds, or only the earliest pottery? Other associated materials should be discussed or tabulated. | We clarified that the six Huarabí sherds are the diagnostic Formative ceramic sample relevant to the present argument, not the totality of project-wide material culture. The broader project recorded other ceramics, but the ceramics associated with geoglyphs elsewhere belong to later periods and were not used as chronological anchors for the present Formative-focused discussion. | Added explicit text stating that ceramics associated with geoglyphs elsewhere in the broader project area belong to later periods. Provided the diagnostic Huarabí ceramic catalogue in Supplementary Table S4. |
| If Pichausa has no Formative pottery, on what basis is it treated as Formative? | We agreed that the earlier wording was too strong. The revised manuscript now states explicitly that Pichausa remains chronologically provisional. Its tentative attribution is based only on morphology, not on independent ceramic or chronometric evidence. | Reframed Pichausa chronology as provisional in the Abstract, Introduction, ceramic section, Discussion, and Conclusions. |
| How are the roads/routes dated? If they are not contemporaneous, what interpretive value do they have? | We now address this directly. The route layers are treated as visible movement proxies in the present landscape, not as securely dated ancient roads or complete reconstructions of past circulation. Their value is analytical: they provide explicit reference networks against which observed geoglyph–route distances can be compared with chance-placement expectations under stated availability definitions. | Revised the Methods to define the route layers as present-landscape proxies, to clarify that they are not independently dated, and to state that the results are interpreted separately by quebrada because route representations differ between them. |
| The six sherds should be placed within the broader settlement history of the middle valley. | We agree. The revised manuscript now explicitly locates Huarabí and Pichausa within a middle-valley landscape that includes nearby Formative sites and U-shaped ceremonial architecture, so the six Huarabí sherds are no longer read in isolation. | Added a broader Formative-period contextual section, including reference to U-shaped buildings and nearby Formative sites in the valley. |
| The Huarabí association could be used to think about a broader Formative sphere of activity rather than only comparing the two quebradas. | We accept this recommendation. The revised discussion now treats Huarabí primarily as a geoglyph locality embedded within a broader Formative archaeological landscape. The cross-quebrada contrast remains analytically useful, but it is no longer the sole cultural payoff of the article. | Rewrote the chronology and cultural-placement discussion so that Huarabí is framed as evidence of Formative-period activity in the vicinity rather than as direct geoglyph dating. |
| Possible shortcut or movement-corridor functions of the quebradas should be considered. | We found this suggestion useful and incorporated it conceptually into the revised discussion. We do not claim that the current data prove a shortcut function, but we now leave open alternatives such as threshold marking, encounter structuring, signaling, and broader organization of movement through quebrada corridors. | Expanded the Discussion to distinguish movement channeling from specific route association and to keep multiple behavioral interpretations open. |
| The manuscript should cite broader comparative literature, including recent work farther south in Peru and the new Stanish et al. article. | Done. The Introduction now includes a broader comparative literature frame and explicitly cites more recent Andean geoglyph scholarship, including the Stanish et al. synthesis. | Expanded the comparative literature in the Introduction and integrated it into the revised Discussion. |

**Reviewer 2**

| **Reviewer comment** | **Response** | **Change made in revised manuscript** |
| --- | --- | --- |
| The paper must state clearly that the earlier *Antiquity* article was the broader overview and that this is a distinct, narrower analysis. | We agree. This is now stated explicitly at the start of the Introduction so that the relationship between the two papers is transparent immediately. | Added a new opening paragraph clarifying that the earlier paper was the broader survey overview and that the present manuscript is a narrower analytical study focused on Huarabí and Pichausa. |
| The manuscript contains too much jargon. | We agree and carried out a full prose-tightening pass. Technical precision was retained where necessary, but repeated cautionary phrasing, overly procedural wording, and dense formulations were reduced. | Revised the Abstract, Introduction, Methods, and first half of the Discussion for clarity and compression. |
| The literature review should extend beyond Nasca-Palpa and include broader Andean comparative material. | We agree. The revised Introduction now includes broader Andean geoglyph literature and uses it to frame geoglyphs not only as route markers but as movement-organized spaces of congregation, performance, and aggregation. | Expanded the literature review to include comparative work from other Andean regions and recent synthetic discussions. |
| It is essential to clarify whether the survey was route-focused, otherwise route proximity could be an artifact of survey design. | We agree completely. The revised manuscript now states explicitly that the survey was regional in scope and not restricted to route corridors. At the same time, we now acknowledge that quantitative effort surfaces are unavailable and that within-polygon detectability may still have varied. | Expanded *Field survey and recording* to describe the survey design as regional rather than route-targeted and to state that the results are interpreted conditional on documented coverage and possible within-polygon effort heterogeneity. |
| The pottery sample is very small, so the chronological argument should be much more cautious. | We agree. The revised manuscript no longer uses the Huarabí ceramics to imply direct geoglyph dating. Instead, they are framed as evidence of Formative-period activity in the vicinity of one geoglyph locality. | Reframed the ceramic discussion, the Abstract, the Discussion, and the Conclusions accordingly. |
| The geoglyph localities should be tied more explicitly to nearby U-shaped buildings and sites. | We accepted this recommendation and expanded the archaeological context accordingly. | Added explicit reference to the U-shaped buildings of Chocas and Pucará and to the concentration of nearby Formative sites in the middle valley. |

**Reviewer 3**

| **Reviewer comment** | **Response** | **Change made in revised manuscript** |
| --- | --- | --- |
| The framework is promising, but inferential scope is too strong for the sample size and the nature of the data. | We agree and narrowed the claims substantially. The revised manuscript now frames the results as differences from tested null models under stated assumptions, not as proof of a single behavioral mechanism or deliberate route-oriented placement. | Revised the title, Discussion, and Conclusions to soften the inferential language and emphasize model dependence. |
| The manuscript should emphasize effect sizes, sensitivity, and model dependence rather than threshold significance. | We agree. The revision now foregrounds availability sensitivity, Δmedian behavior, and the exploratory character of sensitivity analyses rather than treating p-values as standalone substantive findings. | Reworked the Results and Discussion so that the buffer sweep, geomorphic windows, and k-sensitivity are clearly treated as sensitivity diagnostics. |
| The absence of a full terrain-feasibility model is a major limitation. | We agree and now say so directly. The revised manuscript states plainly that the study does not include a bare-earth DTM and does not model slope, curvature, roughness, substrate stability, or other terrain variables directly. | Added explicit acknowledgment that the geomorphic scenarios are exploratory and not substitutes for terrain-based feasibility modeling. |
| Spatial accuracy was underdocumented; GSD/RMSE/GCP quality should be acknowledged. | We agree. The revised Methods now include a dedicated paragraph stating that formal RPAS spatial-accuracy metadata are unavailable and that the analyses should therefore be read as broad-scale comparisons rather than as high-precision locational tests. | Added a dedicated RPAS-accuracy limitation paragraph in the Methods. |
| The manuscript sometimes conflates topographic movement constraints with association to specific routes. | We agree and rewrote the relevant discussion. The revised manuscript now distinguishes general channeling by quebrada topography from hypothesized association with mapped route layers. | Revised the Discussion so that geomorphological channeling and route association are treated as distinct possibilities rather than interchangeable explanations. |
| Ceramic chronology should be framed strictly as evidence of Formative activity nearby, not direct geoglyph dating. | We agree completely and revised the paper in exactly this direction. Huarabí is now framed as evidence of Formative-period activity in the vicinity of one geoglyph locality, not as direct proof of construction age. | Revised the Abstract, ceramic section, Discussion, and Conclusions accordingly. |
| The original version lacked explicit ethics/permitting information. | We agree this was an important omission and corrected it fully. | Added a dedicated ethics statement specifying Ministry of Culture Director’s Resolution No. 00276-2021-DCIA/MC and authors’ COARPE/RNA status. |
| The paper should be reframed as exploratory and model-conditional. | We accepted this recommendation in full and made it one of the main organizing principles of the revision. | The revised manuscript repeatedly states that the results are exploratory, model-conditional, and sensitive to the stated availability assumptions. |

We are grateful for the reviewers’ comments, which improved the manuscript substantially. The revised version now makes a narrower but more defensible argument: Huarabí provides evidence of Formative-period activity in the vicinity of one geoglyph locality, Pichausa remains chronologically provisional, mapped routes are treated as present-landscape proxies, and the statistical results are interpreted as model-conditional differences from tested null models rather than as definitive evidence of a single cultural mechanism.

Sincerely,

Angel Sánchez-Borjas & Christian Mesía Montenegro
