# Supplementary material for: Geoglyphs and formative-period activity in the middle Chillón Valley, Peru: Ceramic association and null-model tests of route proximity
Source: PLoS One. 2026 Jun 8;21(6):e0350855. doi: 10.1371/journal.pone.0350855 (PMC13245780; doi:10.1371/journal.pone.0350855)
Supplement: Attachment — Submitted filename: Letter to reviewers v.2.docx [file pone.0350855.s015.docx]

Lima, May 8^th^, 2026

Dear Dr. Reepmeyer,

Academic Editor
PLOS ONE

We thank you and the reviewers for the careful assessment of our revised manuscript. We have made a further revision focused on the remaining issues raised in this round: the tentative character of the results, the small empirical sample, the limits of the availability model, and the need to present the contribution as exploratory and methodological rather than as a substantive cultural explanation.

The manuscript now frames the results consistently as deviations from specified null models, not as evidence of intentional route association, construction chronology, or valley-wide spatial organization. We also clarify that the Huarabí ceramics indicate nearby Formative-period activity but do not directly date the geoglyphs, and that Pichausa remains chronologically unresolved. Additional revisions address spatial uncertainty, route proxies, survey-effort uncertainty, and the absence of terrain-based construction-feasibility modeling.

Our responses are provided below.

**Response to the Academic Editor**

**Comment:**
“Please review the comments closely, particularly of Reviewer 2 [Reviewer 3 in the comments below] in reference to the tentative nature of the results and the relative small size of the sample dataset.”

**Response:**
We have revised the manuscript to make the tentative scope of the analysis explicit. The Abstract now states the empirical scale of the study—four geoglyph groups and six Huarabí ceramic findspots—and specifies that the results do not establish intentional route association, construction age, or valley-wide spatial organization. The Conclusions have also been revised to state that the findings should be read as exploratory, model-dependent evidence of deviation from tested null models.

We strengthened the limitations section by emphasizing the small sample, the restriction of inference to the recorded contexts, and the fact that the analysis should not be generalized to the entire valley. We also added clearer language explaining that the availability windows are geometric proxies rather than terrain-based models of construction feasibility.

**General comment**

**Reviewer 3:**
“The revised manuscript significantly enhances transparency, interpretive caution, and adherence to ethical standards. The authors have thoughtfully addressed the previous review and have commendably reframed their claims to be model-conditional and exploratory instead of deterministic. However, some key methodological issues, especially in terrain-based feasibility modeling, remain unresolved, limiting the interpretive value of the analysis. Although the manuscript has improved significantly, it still needs specific clarifications and a softer presentation of its conclusions before publication.”

**Response:**
We thank Reviewer 3 for recognizing the improvements in transparency, interpretive caution, and ethical reporting. We agree that the absence of terrain-based feasibility modeling limits the interpretive scope of the analysis. We have therefore revised the Abstract, Methods, Discussion, and Conclusions to make this limitation more explicit.

The revised manuscript now states that the availability windows are geometric approximations rather than independently modeled construction-feasibility surfaces. It also specifies that observed deviations may reflect cultural siting preferences, geomorphic constraints, visibility conditions, survey-process effects, or misspecification of the counterfactual placement space. The Conclusions have been softened accordingly.

**Model-dependent inference and “structured placement”**

**Reviewer 3:**
“Some language still implies a stronger notion of ‘structured placement’ than the data can support, especially considering the dataset includes only four geoglyph units and six ceramic findspots. The results should be uniformly described as deviations from specific tested null models rather than as proof of broader spatial structuring in archaeology.”

**Response:**
We have removed language that could be read as claiming broader spatial structuring. The manuscript now describes the results as deviations from tested null models under stated availability assumptions. Terms such as “structured placement” have been replaced with more precise language, including “model-dependent spatial discrepancy,” “deviation from the tested null model,” and “difference between observed and simulated distance distributions.”

The Discussion now states that the results support only a model-dependent spatial discrepancy in Huarabí, not intentional route association or broader spatial organization. The Conclusions likewise state that the findings are exploratory and model-dependent, rather than evidence for a single cultural mechanism.

**Terrain-based feasibility modeling**

**Reviewer 3:**
“The main issue persists: feasibility is still not directly modeled, and the null model assumes spatial availability that may not reflect true construction constraints in quebrada landscapes. Consequently, observed deviations might partly result from misspecification of the counterfactual rather than genuine spatial patterns.”

**Response:**
We have expanded this limitation in the Methods. The manuscript now states that the availability windows are geometric approximations of potential placement space rather than independently modeled construction-feasibility surfaces. We specify that the analysis does not incorporate bare-earth DTM-derived slope, microtopography, substrate, surface clast density, visibility of cleared lines, erosion history, or geomorphic stability.

We did not add a new terrain-based feasibility model because the required bare-earth terrain and substrate data are not available for the present dataset. Instead, we have restricted the interpretation and identify terrain-based feasibility modeling as a priority for future work.

**Spatial accuracy and uncertainty**

**Reviewer 3:**
“Spatial uncertainty might be comparable to some observed differences, emphasizing the exploratory and non-deterministic nature of the findings.”

**Response:**
We added explicit language on spatial uncertainty. The Methods now state that formal spatial-accuracy metadata, including ground sampling distance, georeferencing RMSE, and ground-control-point error statistics, are unavailable for the present dataset. We also note that, because some reported contrasts occur at relatively short distance scales, positional uncertainty may be non-trivial relative to the smaller observed differences.

This clarification reinforces the treatment of the analysis as a broad-scale spatial comparison, not a high-precision test of exact locational accuracy.

**Movement constraints versus route association**

**Reviewer 3:**
“The revision significantly improves the distinction between movement constraints and route association. However, while the conceptual separation is well established, the analysis still lacks independent terrain-based modeling, preventing a clear disentangling of geomorphology’s role from potential cultural siting preferences.”

**Response:**
We have retained the distinction between general movement constraints imposed by quebrada topography and proximity to mapped route proxies. We also added stronger caution that these factors cannot be separated with the present data.

The Discussion now states that apparent route proximity may partly reflect geomorphological channeling and survey process rather than intentional siting relative to circulation corridors. The future-work section identifies terrain-based feasibility modeling, visibility analysis, and standardized route representations as necessary steps for distinguishing cultural siting practices from geomorphic constraints and survey effects.

**Chronology and ceramic associations**

**Reviewer 3:**
“The revision could be improved by refining the language to emphasize that ceramics are only useful as contextual indicators of local activity, not as definitive markers of chronological or cultural placement.”

**Response:**
We revised the treatment of the ceramic evidence throughout the manuscript. The Abstract now states that diagnostic Formative-period ceramics were recorded only in Huarabí, where they indicate nearby activity rather than directly dating geoglyph construction. The ceramic-analysis section treats ceramics as a coarse typological attribution based on surface material, not as a basis for fine-grained phasing or direct dating.

The Discussion now states that Huarabí provides the only currently documented contextual indication of nearby Formative-period activity, and that the depositional relationship between the sherds and the linework cannot be established. We also clarify that Pichausa is not assigned a secure Formative chronology; its irregular morphology is treated only as a comparative observation.

**Conceptual contribution and interpretive framing**

**Reviewer 3:**
“Framing the main contribution more explicitly as methodological and exploratory, rather than as offering a substantive cultural explanation, would enhance the manuscript.”

**Response:**
We have revised the framing accordingly. The Abstract now identifies the principal contribution as methodological: the study demonstrates how small geoglyph datasets can be evaluated through transparent null-model procedures while making explicit the limits imposed by sample size, surface chronology, spatial uncertainty, and the absence of terrain-based feasibility modeling.

The Discussion now treats the Huarabí result as a basis for further testing rather than as a substantive explanation. The final future-work paragraph has also been revised to state that future research should test whether the observed proximity patterns reflect cultural siting practices, geomorphic constraints, visibility conditions, survey-process effects, or some combination of these factors.

We thank you and both reviewers for their detailed comments. The revised manuscript now presents the analysis as exploratory, model-dependent, and methodologically focused. It avoids stronger cultural claims that are not supported by the present sample and clarifies the limits imposed by surface chronology, spatial uncertainty, route proxies, survey-effort uncertainty, and the absence of terrain-based feasibility modeling.

Sincerely,

Christian Mesía-Montenegro & Angel Sánchez-Borjas
