# Supplementary material for: Geoglyphs and formative-period activity in the middle Chillón Valley, Peru: Ceramic association and null-model tests of route proximity
Source: PLoS One. 2026 Jun 8;21(6):e0350855. doi: 10.1371/journal.pone.0350855 (PMC13245780; doi:10.1371/journal.pone.0350855)
Supplement: Peer review history

# Peer review: Decision Letter 0

**DOI:** 10.1371/journal.pone.0350855.r001

**Authors:** Christian Reepmeyer

1 Apr 2026

PONE-D-26-03379

Formative-period geoglyphs in the middle Chillón Valley, Peru: survey documentation, ceramic association, and null-model tests of route proximity

PLOS One

Dear Dr. Mesia-Montenegro,

Thank you for submitting your manuscript to PLOS ONE. After careful consideration, we feel that it has merit but does not fully meet PLOS ONE’s publication criteria as it currently stands. Therefore, we invite you to submit a revised version of the manuscript that addresses the points raised during the review process.

The paper makes a substantial methodological contribution to the field, please review the comments made carefully. However, there are a few issues raised by the reviewers, which needs to be addressed prior to a possible publication:

1. There is a disconnect between the interpretative claims and the evidence base. Either rewrite the interpretative claims in a more tentative fashion or increase the evidence base.

2. Acknowledge missing features in the analysis, for example the absence of terrain-based feasibility modeling and its implications for inference

3. As the sites are surface finds, please review the chronological claims made

4. Provide adequate information on research permits

Please submit your revised manuscript by May 16 2026 11:59PM. If you will need more time than this to complete your revisions, please reply to this message or contact the journal office at plosone@plos.org. When you're ready to submit your revision, log on to https://www.editorialmanager.com/pone/ and select the 'Submissions Needing Revision' folder to locate your manuscript file.

Please include the following items when submitting your revised manuscript:

- A letter that responds to each point raised by the academic editor and reviewer(s). You should upload this letter as a separate file labeled 'Response to Reviewers'.
- A marked-up copy of your manuscript that highlights changes made to the original version. You should upload this as a separate file labeled 'Revised Manuscript with Track Changes'.
- An unmarked version of your revised paper without tracked changes. You should upload this as a separate file labeled 'Manuscript'.

If you would like to make changes to your financial disclosure, please include your updated statement in your cover letter. Guidelines for resubmitting your figure files are available below the reviewer comments at the end of this letter.

If applicable, we recommend that you deposit your laboratory protocols in protocols.io to enhance the reproducibility of your results. Protocols.io assigns your protocol its own identifier (DOI) so that it can be cited independently in the future. For instructions see: https://journals.plos.org/plosone/s/submission-guidelines#loc-laboratory-protocols. Additionally, PLOS ONE offers an option for publishing peer-reviewed Lab Protocol articles, which describe protocols hosted on protocols.io. Read more information on sharing protocols at https://plos.org/protocols?utm_medium=editorial-email&utm_source=authorletters&utm_campaign=protocols.

As the corresponding author, your ORCID iD is verified in the submission system and will appear in the published article. PLOS supports the use of ORCID, and we encourage all coauthors to register for an ORCID iD and use it as well. Please encourage your coauthors to verify their ORCID iD within the submission system before final acceptance, as unverified ORCID iDs will not appear in the published article. *Only* the individual author can complete the verification step; PLOS staff *cannot* verify ORCID iDs on behalf of authors.

We look forward to receiving your revised manuscript.

Kind regards,

Christian Reepmeyer, PhD

Academic Editor

PLOS One

Journal Requirements:

When submitting your revision, we need you to address these additional requirements.

1. Please ensure that your manuscript meets PLOS ONE's style requirements, including those for file naming. The PLOS ONE style templates can be found at

https://journals.plos.org/plosone/s/file?id=wjVg/PLOSOne_formatting_sample_main_body.pdf and https://journals.plos.org/plosone/s/file?id=ba62/PLOSOne_formatting_sample_title_authors_affiliations.pdf

2. In your manuscript, please provide additional information regarding the specimens used in your study. Ensure that you have reported human remain specimen numbers and complete repository information, including museum name and geographic location.

If permits were required, please ensure that you have provided details for all permits that were obtained, including the full name of the issuing authority, and add the following statement:

'All necessary permits were obtained for the described study, which complied with all relevant regulations.'

If no permits were required, please include the following statement:

'No permits were required for the described study, which complied with all relevant regulations.'

For more information on PLOS One's requirements for paleontology and archeology research, see https://journals.plos.org/plosone/s/submission-guidelines#loc-paleontology-and-archaeology-research.

3. Thank you for stating the following financial disclosure:

Field research was funded by the Centro de Investigaciones Arqueológicas Precolombinas (CIAP). Formal analyses were funded by Universidad Privada del Norte (UPN).

Please state what role the funders took in the study. If the funders had no role, please state: "The funders had no role in study design, data collection and analysis, decision to publish, or preparation of the manuscript."

If this statement is not correct you must amend it as needed.

Please include this amended Role of Funder statement in your cover letter; we will change the online submission form on your behalf.

4. Please ensure that you refer to Figure 1 – 4 in your text as, if accepted, production will need this reference to link the reader to the figure.

5. We note that Figure(s) 1, 2, 3 in your submission contain [map/satellite] images which may be copyrighted. All PLOS content is published under the Creative Commons Attribution License (CC BY 4.0), which means that the manuscript, images, and Supporting Information files will be freely available online, and any third party is permitted to access, download, copy, distribute, and use these materials in any way, even commercially, with proper attribution. For these reasons, we cannot publish previously copyrighted maps or satellite images created using proprietary data, such as Google software (Google Maps, Street View, and Earth). For more information, see our copyright guidelines: http://journals.plos.org/plosone/s/licenses-and-copyright.

We require you to either (1) present written permission from the copyright holder to publish these figures specifically under the CC BY 4.0 license, or (2) remove the figures from your submission:

a. You may seek permission from the original copyright holder of Figure(s) 1, 2, 3 to publish the content specifically under the CC BY 4.0 license.

We recommend that you contact the original copyright holder with the Content Permission Form (http://journals.plos.org/plosone/s/file?id=7c09/content-permission-form.pdf) and the following text:

“I request permission for the open-access journal PLOS ONE to publish XXX under the Creative Commons Attribution License (CCAL) CC BY 4.0 (http://creativecommons.org/licenses/by/4.0/). Please be aware that this license allows unrestricted use and distribution, even commercially, by third parties. Please reply and provide explicit written permission to publish XXX under a CC BY license and complete the attached form.”

Please upload the completed Content Permission Form or other proof of granted permissions as an "Other" file with your submission.

In the figure caption of the copyrighted figure, please include the following text: “Reprinted from [ref] under a CC BY license, with permission from [name of publisher], original copyright [original copyright year].”

b. If you are unable to obtain permission from the original copyright holder to publish these figures under the CC BY 4.0 license or if the copyright holder’s requirements are incompatible with the CC BY 4.0 license, please either i) remove the figure or ii) supply a replacement figure that complies with the CC BY 4.0 license. Please check copyright information on all replacement figures and update the figure caption with source information. If applicable, please specify in the figure caption text when a figure is similar but not identical to the original image and is therefore for illustrative purposes only.

The following resources for replacing copyrighted map figures may be helpful:

USGS National Map Viewer (public domain): http://viewer.nationalmap.gov/viewer/

The Gateway to Astronaut Photography of Earth (public domain): http://eol.jsc.nasa.gov/sseop/clickmap/

Maps at the CIA (public domain): https://www.cia.gov/library/publications/the-world-factbook/index.html and https://www.cia.gov/library/publications/cia-maps-publications/index.html

NASA Earth Observatory (public domain): http://earthobservatory.nasa.gov/

Landsat: http://landsat.visibleearth.nasa.gov/

USGS EROS (Earth Resources Observatory and Science (EROS) Center) (public domain): http://eros.usgs.gov/#

Natural Earth (public domain): http://www.naturalearthdata.com/

6. We note that Figure S4 in your submission contain copyrighted images. All PLOS content is published under the Creative Commons Attribution License (CC BY 4.0), which means that the manuscript, images, and Supporting Information files will be freely available online, and any third party is permitted to access, download, copy, distribute, and use these materials in any way, even commercially, with proper attribution. For more information, see our copyright guidelines: http://journals.plos.org/plosone/s/licenses-and-copyright.

We require you to either (1) present written permission from the copyright holder to publish these figures specifically under the CC BY 4.0 license, or (2) remove the figures from your submission:

a. You may seek permission from the original copyright holder of Figure S4 to publish the content specifically under the CC BY 4.0 license.

We recommend that you contact the original copyright holder with the Content Permission Form (http://journals.plos.org/plosone/s/file?id=7c09/content-permission-form.pdf) and the following text:

“I request permission for the open-access journal PLOS ONE to publish XXX under the Creative Commons Attribution License (CCAL) CC BY 4.0 (http://creativecommons.org/licenses/by/4.0/). Please be aware that this license allows unrestricted use and distribution, even commercially, by third parties. Please reply and provide explicit written permission to publish XXX under a CC BY license and complete the attached form.”

Please upload the completed Content Permission Form or other proof of granted permissions as an "Other" file with your submission.

In the figure caption of the copyrighted figure, please include the following text: “Reprinted from [ref] under a CC BY license, with permission from [name of publisher], original copyright [original copyright year].”

b. If you are unable to obtain permission from the original copyright holder to publish these figures under the CC BY 4.0 license or if the copyright holder’s requirements are incompatible with the CC BY 4.0 license, please either i) remove the figure or ii) supply a replacement figure that complies with the CC BY 4.0 license. Please check copyright information on all replacement figures and update the figure caption with source information. If applicable, please specify in the figure caption text when a figure is similar but not identical to the original image and is therefore for illustrative purposes only.

7. Please include captions for your Supporting Information files at the end of your manuscript, and update any in-text citations to match accordingly. Please see our Supporting Information guidelines for more information: http://journals.plos.org/plosone/s/supporting-information.

8. If the reviewer comments include a recommendation to cite specific previously published works, please review and evaluate these publications to determine whether they are relevant and should be cited. There is no requirement to cite these works unless the editor has indicated otherwise.

[Note: HTML markup is below. Please do not edit.]

Reviewers' comments:

Reviewer's Responses to Questions

**Comments to the Author**

1. Is the manuscript technically sound, and do the data support the conclusions?

The manuscript must describe a technically sound piece of scientific research with data that supports the conclusions. Experiments must have been conducted rigorously, with appropriate controls, replication, and sample sizes. The conclusions must be drawn appropriately based on the data presented.

Reviewer #1: Partly

Reviewer #2: Yes

Reviewer #3: Partly

**********

2. Has the statistical analysis been performed appropriately and rigorously?

Reviewer #1: Yes

Reviewer #2: Yes

Reviewer #3: Yes

**********

3. Have the authors made all data underlying the findings in their manuscript fully available?

The PLOS Data policy requires authors to make all data underlying the findings described in their manuscript fully available without restriction, with rare exception (please refer to the Data Availability Statement in the manuscript PDF file). The data should be provided as part of the manuscript or its supporting information, or deposited to a public repository. For example, in addition to summary statistics, the data points behind means, medians and variance measures should be available. If there are restrictions on publicly sharing data—e.g. participant privacy or use of data from a third party—those must be specified.

Reviewer #1: Yes

Reviewer #2: Yes

Reviewer #3: No

**********

4. Is the manuscript presented in an intelligible fashion and written in standard English?

PLOS ONE does not copyedit accepted manuscripts, so the language in submitted articles must be clear, correct, and unambiguous. Any typographical or grammatical errors should be corrected at revision, so please note any specific errors here.

Reviewer #1: Yes

Reviewer #2: No

Reviewer #3: No

**********

5. Review Comments to the Author

Please use the space provided to explain your answers to the questions above. You may also include additional comments for the author, including concerns about dual publication, research ethics, or publication ethics. (Please upload your review as an attachment if it exceeds 20,000 characters)

Reviewer #1: Overall, there are some very interesting questions that can be considered by placing these geoglyphs into a landscape and cultural context. The analysis here is transparent in its methods and expectations, which is a positive thing, but it feels like the small dataset could be stronger if it were integrated into a broader archaeological context, rather than abstracted through a simulation that feels like it lacks some key information.

Lines 184-185: Given the number of geoglyphs encountered in the project, it would be helpful to know how the two quebradas discussed here were selected for analysis.

Line 196ff.: Were the six Formative sherds the only surface finds recorded by the field research, or just the earliest pottery? If there were other artifacts (later ceramic phases, preceramic lithics), it would be good to tabulate those and to discuss the interpretation of those collections. If these were the only artifacts encountered, then it would be good to state that clearly.

Lines 218-226: Given that no Formative pottery was reported for Pichausa, it would be helpful to know how the geoglyphs in that quebrada were identified as Formative.

Lines 230ff.: How is the chronology of roads/routes determined? If these are not validated as contemporaneous with the construction of the geoglyphs, what interpretive value do they have in the modeling exercise? Since these are not all of the existing routes, how were these selected?

Line 441ff.: It would be helpful to have a broader context for the settlement history of this part of the valley, either through research by the authors and/or through reference to Silva’s regional survey. If there are Formative settlements nearby, that might complicate arguments that the 6 sherds encountered are associated with the earliest construction of the geoglyphs.

Lines 446-448: If one of the two study areas is chronologically inconclusive, then the interpretive power of the analysis seems restricted to general inferences about the extent to which geoglyph construction was done in proximity to low-cost routes. I worry about the potential for circularity here, since the study does not incorporate all known or possible routes in the area. It would be interesting to consider some alternatives to the siting of geoglyphs, such as overall visibility or location along routes between known Formative sites.

Lines 476ff.: The point about Huarabi’s distinctive association with Formative pottery seems like it could be used productively to consider the Formative “sphere of activity” referenced here, which would probably require multiscalar discussion of settlement patterns, landscapes of movement, and activity patterns that could be related to the geoglyphs there. The cultural question seems more compelling than the comparison of the two quebradas, given the limited inferences that can be made with the other geoglyphs.

Lines 482ff.: If movement is relatively channeled through these quebradas, what purpose do the geoglyphs play? On the map it looks like the quebradas might be a shortcut to avoid a meandering curve in the main valley, presumably used by people moving between regions.

It might be worthwhile to mention some of the geoglyph work farther south in Peru in the introduction, and there is a new article by Stanish et al. that might help to articulate a stronger cultural argument:

Stanish, Charles, Henry Tantaleán, Carito Tavera, Jacob Bongers, and Karen Wise. "A Theory of Andean Geoglyphs." Journal of Anthropological Research 82, no. 1 (2026): 000-000.

NB: My analytical expertise does not extend to the kind of simulation modeling presented here, and the journal is encouraged to consult a reviewer who can address the design and execution of that part of the study.

Reviewer #2: This is a good paper and interesting contribution to the literature on geoglyphs. I suggest publication with some modifications at the discretion of the authors and editor.

First the authors must add an explicit statement in the beginning noting tht the Antiquity piece previously published by the authors describes the broader survey project. It is the overview publication. Then note that this manuscript analyzes a specific set of data from this larger project. This manuscript adds a new analysis which is a Monte Carlo null-model inference and sensitivity analysis.

The paper has an overabundance of jargon. A native English-speaking editor should clean this up.

The statistical analysis seems to be sound and appropriate. I see no serious issues but a statistician should be consulted.

Line 106. “The Nasca-Palpa corpus dominates the international literature”. This is true but there are other published works on geoglyphs in Arequipa, Casma, Chincha and northern Chile. These should be briefly noted to provide a fuller research context. This paper in particular seems very relevant to this manuscript: Bikoulis, P., Gonzalez-Macqueen, F., Spence-Morrow, G., Bautista, S., Alvarez, W.Y. and Jennings, J., 2018. Ancient pathways and geoglyphs in the Sihuas Valley of southern Peru. Antiquity, 92(365), pp.1377-1391

It is very important that the authors expand on the survey methodology. If this was a full regional coverage methodology that included modern and presumed earlier routes as welll as areas away from these routes, the interpretation is strongly supported. However, if the pedestrian survey overly weighted these routes, then this could inflate apparent route proximity. Figure 2 seems to suggest that the survey was focused on pathways since they tend to follow gullies. These pathways should be included in the map. This is an issue that has to be addressed. Did the drone survey look for geoglyphs outside of the pathways? Clearly, if there was no systematic survey away from the routes then the interpretation is weakened because the methodology insures that only geoglyphs near the roads were studied. On the other hand, if the Monge Carlo null model took that into consideration, the authors need to be forcefully explain this.

The sample of formative pottery to date the geoglyphs is extremely low. One should recall that some of the earliest work on the Nazca geoglyphs claim that they were LIP in date based upon diagnostic pottery found on the surface on or near the geoglyphs. Subsequent work indicated that this was not the case. It's very difficult to date geoglyphs, I understand this and the discussion in lines 476 – 481 should be highlighted. In that regard, the authors need to demonstrate some geographical linkage to Formative sites, even if these are far away but on the travel routes documented for the geoglyphs. In this regard, lines 160 -167 in the manuscript should explicitly tie the geoglyphs to the U shaped buildings in the valley. Furthermore, if there are other sites from different time periods in the valley then these should be mentioned.

Reviewer #3: This manuscript presents previously unpublished geoglyph contexts from two quebradas of the middle Chillón Valley and applies explicit counterfactual (Monte Carlo) spatial models to evaluate their relationship to mapped circulation features. The analytical framework is methodologically sound and, in principle, well suited to evaluating hypotheses about non-random spatial relationships between geoglyphs and circulation features. The authors are also commendably cautious in distinguishing statistical deviation from cultural interpretation.

That said, several substantial issues must be addressed before the manuscript can be considered suitable for publication in PLOS ONE. These issues concern the inferential scope of the analysis, the adequacy of the spatial data and landscape modeling, chronological interpretation, and compliance with research ethics and permitting requirements.

1. Inferential scope, sample size, and statistical interpretation

Despite careful caveating, the Discussion and Conclusions still over-interpret results derived from an extremely small empirical sample. The Formative dataset comprises only four geoglyph units (two per quebrada), supplemented in Huarabí by six surface ceramic findspots. Under these conditions, Monte Carlo p-values (e.g., p = 0.026 under corridor-restricted availability) cannot be treated as substantive evidence for “structured” placement. At most, the results demonstrate a discrepancy between observed and simulated distance distributions under a single, highly specific operationalization of availability and route proxies. The non-monotonic behavior observed in the buffer sweep—including sign reversals in Δmedian—clearly indicates that inference is highly sensitive to how feasible placement space is defined. This instability substantially weakens any behavioral interpretation. The manuscript should therefore be reframed more explicitly as exploratory and methodological, with emphasis placed on effect sizes, sensitivity diagnostics, and model dependence rather than on thresholded statistical significance.

2. Absence of terrain-based feasibility modeling (critical limitation)

Most critically, the analysis operates without a bare-earth Digital Terrain Model (DTM) and without any explicit modeling of landscape feasibility. The study relies on UAV-derived surface models and planimetric representations of geoglyphs and routes, but does not incorporate slope, exposure, microrelief, substrate stability, or geomorphic suitability into either the null models or the definition of feasible placement space. This omission is not a minor technical detail. Geoglyphs cannot be constructed everywhere in a quebrada landscape, and feasible placement space is inherently heterogeneous. Treating all locations within the surveyed polygon (or its buffered expansions) as equally available risks overestimates spatial freedom and fundamentally mis-specifies the counterfactual model against which observed patterns are evaluated. The sensitivity analyses using progressively expanded availability windows demonstrate that inference is highly dependent on how placement space is defined. While the authors correctly describe this as scale dependence, the deeper issue is that window-based buffering is a geometric proxy for feasibility rather than a substantive model of it. Without terrain-based constraints, deviations observed under broader windows may reflect null-model inflation rather than meaningful behavioral structure.

3. Spatial accuracy and data resolution

Interpretation is further limited by the absence of explicit accuracy metrics for the UAV-derived spatial data. No information is provided on ground sampling distance (GSD), georeferencing error (e.g., RMSE), or the use, number, and quality of ground control points. Given that reported effect sizes and median distance differences often operate at the scale of tens of meters, positional uncertainty may be comparable in magnitude to the observed effects. These uncertainties should be quantified or, at least, explicitly bounded.

4. Conceptual separation of movement constraints and route association

The Discussion occasionally conflates two analytically distinct phenomena:

(a) general movement constraints imposed by quebrada topography, and

(b) hypothesized association between geoglyph placement and specific circulation routes.

The former does not, by itself, support the latter. Without explicit modeling of feasibility (e.g., slope, substrate, visibility) independent of mapped routes, proximity to routes may simply reflect geomorphological channeling and survey process rather than intentional placement relative to circulation corridors.

5. Chronology and ceramic associations

The treatment of Formative ceramics in Huarabí is appropriately cautious in places but still leans too far toward cultural placement. Given the small number of surface finds, their utilitarian character, and the absence of stratigraphic association with geoglyph construction, these materials should be framed strictly as evidence for Formative-period activity in the vicinity, not as support for geoglyph dating. Statements suggesting that Huarabí provides a “strong basis” for situating geoglyphs within a Formative sphere should be softened accordingly.

6. Fieldwork authorization and research ethics (major omission)

The manuscript states that a “systematic pedestrian survey” was conducted in 2021–2022, but it does not provide formal information regarding authorization by the Ministry of Culture of the Republic of Peru. In Peru, all archaeological fieldwork—including survey—must be conducted under a formally approved archaeological project supported by:

• a current Resolution (Resolución Directoral) issued by the Dirección de Calificación de Intervenciones Arqueológicas of the Ministry of Culture, and

• active COARPE registration, including valid annual habilitación for the project director.

At present, the manuscript includes only acknowledgements, without providing permit numbers, issuing authority, or confirmation of COARPE status. This is a serious omission. For ethical compliance and transparency, the authors must explicitly state the permit number(s), issuing body, and year(s) of validity, or otherwise clarify the legal framework under which the fieldwork was conducted. Without this information, the manuscript does not meet basic standards of research ethics and legal compliance.

7. Recommendations

I recommend major revision, conditional on the following:

• reframing the analytical claims as explicitly exploratory and model-conditional;

• removing or further softening language that implies patterned or intentional route-related placement;

• explicitly acknowledging the absence of terrain-based feasibility modeling and its implications for inference;

• clarifying the limits of chronological interpretation based on surface ceramics; and

• providing full and explicit documentation of Peruvian archaeological permits and authorizations.

Addressing these points would substantially strengthen the manuscript’s methodological integrity and ensure compliance with both scientific and ethical standards.

**********

6. PLOS authors have the option to publish the peer review history of their article (what does this mean?). If published, this will include your full peer review and any attached files.

If you choose “no”, your identity will remain anonymous but your review may still be made public.

**Do you want your identity to be public for this peer review?** For information about this choice, including consent withdrawal, please see our Privacy Policy.

Reviewer #1: No

Reviewer #2: No

Reviewer #3: No

**********

[NOTE: If reviewer comments were submitted as an attachment file, they will be attached to this email and accessible via the submission site. Please log into your account, locate the manuscript record, and check for the action link "View Attachments". If this link does not appear, there are no attachment files.]

To ensure your figures meet our technical requirements, please review our figure guidelines: https://journals.plos.org/plosone/s/figures

You may also use PLOS’s free figure tool, NAAS, to help you prepare publication quality figures: https://journals.plos.org/plosone/s/figures#loc-tools-for-figure-preparation.

NAAS will assess whether your figures meet our technical requirements by comparing each figure against our figure specifications.

# Author comment: Author response to Decision Letter 1

**DOI:** 10.1371/journal.pone.0350855.r002

13 Apr 2026

Lima, April 9, 2026

Dear Editor and Reviewers,

We thank you for your careful reading of our manuscript and for the thoughtful and constructive recommendations. We revised the paper substantially in response. The revised version now clarifies the relationship of this article to our earlier survey overview, narrows the chronological claim, expands the archaeological framing of Huarabí and Pichausa within the middle Chillón Valley, clarifies the status of mapped routes as present-landscape proxies, states explicitly that the survey was regional rather than route-targeted, makes the inferential limits of the spatial analysis more explicit, adds a dedicated RPAS-accuracy limitation, and includes a formal ethics statement with permit information. We also reorganized the supporting information so that the buffer sweeps, geomorphic scenarios, and k-sensitivity analyses are presented clearly as sensitivity diagnostics rather than as primary inferential results.

Reviewer 1 correctly noted that the dataset is small and that the paper would be more compelling if it were integrated more fully into the broader archaeological landscape of the middle valley. We agreed with that assessment and revised the manuscript accordingly. The revised version now gives much greater weight to the Formative-period setting of the middle Chillón Valley and no longer treats the simulation results as the only or even the principal archaeological payoff of the paper. We added a fuller contextual section on Formative developments on the Andean central coast, incorporated the U-shaped architectural tradition more explicitly, and located Huarabí and Pichausa in relation to nearby Formative sites and middle-valley ceremonial architecture. The discussion now treats Huarabí primarily as a geoglyph locality embedded within a broader Formative archaeological landscape rather than as a narrow statistical case.

This broader reframing also helped us respond to the reviewer’s concern about why Huarabí and Pichausa were selected from the larger survey universe. We now explain directly that the present article does not aim to treat all geoglyphs recorded in the broader project. Rather, it focuses on the two quebradas that contain the irregular geoglyph forms under discussion, with Huarabí additionally important because it yielded diagnostic Formative ceramics in geoglyph-adjacent surface contexts. That clarification is now stated explicitly in the fieldwork section so that the analytical scope of the paper is transparent from the outset.

Reviewer 1 also raised an important question about whether the six Huarabí sherds were the only surface finds or only the earliest pottery. We clarified this point in the revision. The six Huarabí sherds are the diagnostic Formative ceramic sample relevant to the present argument, not the totality of project-wide material culture. The broader project recorded ceramics elsewhere, but the ceramics associated with geoglyphs in other parts of the survey area belong to later periods and were therefore not used as chronological anchors for the present Formative-focused analysis. To make this more transparent, we now state this explicitly in the manuscript and provide the Huarabí diagnostic ceramic catalogue in the supporting information. This keeps the chronological discussion tied to the material that is actually relevant to the argument advanced in the article.

The same reviewer also questioned the basis for treating the Pichausa geoglyphs as Formative in the absence of diagnostic Formative ceramics from that quebrada. We agreed that the earlier wording was too strong and revised it throughout. The revised manuscript now states plainly that Pichausa remains chronologically provisional. Its tentative attribution rests only on morphology, not on ceramic or chronometric evidence. By contrast, Huarabí is treated as the stronger archaeological context because diagnostic Formative ceramics were recorded there in geoglyph-adjacent surface contexts. This chronological asymmetry is now acknowledged directly in the Abstract, Introduction, ceramic section, Discussion, and Conclusions.

Reviewer 1 also pressed us on the status and interpretive value of the mapped routes. We responded by revising the Methods so that the route layers are now defined explicitly as visible movement proxies in the present landscape, not as securely dated ancient roads or as complete reconstructions of past circulation. Their analytical value is therefore limited but explicit: they provide reference networks against which observed geoglyph–route distances can be compared with chance-placement expectations under stated availability definitions. We also now state clearly that route representations differ between Huarabí and Pichausa and that all inferential tests are therefore carried out separately by quebrada rather than pooled into a single valley-wide test. This revision was intended to address both the chronology issue and the possibility of circularity in the route-based interpretation.

The reviewer’s suggestion that Huarabí should be interpreted within a broader Formative “sphere of activity,” and not simply as one side of a two-quebrada contrast, was especially helpful. We followed that recommendation closely. The revised discussion now emphasizes that the Huarabí ceramics do not directly date the geoglyphs. Instead, they place one geoglyph locality within a middle-valley landscape that also includes nearby Formative sites and U-shaped ceremonial architecture. This is now the primary archaeological significance of Huarabí in the paper. The cross-quebrada contrast remains analytically useful, but it is no longer the only or strongest cultural interpretation. In the same spirit, we incorporated the reviewer’s observation that the quebradas may have functioned as movement corridors or shortcuts within the valley. We do not claim that the present data can prove that specific behavioral function, but the revised discussion now explicitly leaves open alternatives such as threshold marking, encounter structuring, signaling, or the broader organization of movement through quebrada space. Finally, we expanded the comparative literature in response to the reviewer’s request, including broader Andean scholarship and recent synthetic work on geoglyphs as movement-organized spaces rather than only route markers.

Reviewer 2’s comments led us to improve the paper’s clarity and framing substantially. We agreed that the manuscript had to state clearly that the earlier Antiquity article was the broader overview publication and that the present paper is a distinct, narrower analytical study. The revised Introduction now says this directly in the opening paragraph. We also agreed with the reviewer that the earlier version contained too much jargon. In response, we carried out a full prose-tightening pass across the Abstract, Introduction, Methods, and the first half of the Discussion. The goal was not to remove technical precision, but to reduce repeated cautionary phrasing, overly procedural wording, and unnecessary density. We also broadened the literature review beyond Nasca-Palpa to include a more representative Andean comparative framework, as the reviewer recommended.

Reviewer 2 also raised an important concern about possible route-weighting in the survey design. We agreed entirely and revised the fieldwork section to state explicitly that the survey was regional in scope and not restricted to route corridors. It included areas containing modern circulation features, areas with presumed earlier routes, and sectors away from both. At the same time, we now acknowledge that quantitative effort surfaces are unavailable and that within-polygon detectability may still have varied. We therefore interpret the null-model results as departures from chance expectations conditional on documented coverage and potential within-polygon heterogeneity in detectability, not as proof that circulation independently structured geoglyph placement. This clarification was central to the revision. The reviewer’s parallel concern about the very small pottery sample also led us to further tighten the chronological framing, so that the Huarabí ceramics are now consistently presented as evidence of Formative-period activity in the vicinity of one geoglyph locality, not as direct geoglyph dating. In response to the reviewer’s final major point, we also tied the geoglyph localities more explicitly to nearby U-shaped buildings and Formative sites in the middle valley.

Reviewer 3’s comments were especially important for sharpening the inferential discipline of the paper. We agreed that the original version overstated the scope of what could be inferred from a small and chronologically limited dataset. In response, we narrowed the title, softened the central claims, and rewrote key parts of the Discussion and Conclusions so that the results are now framed as differences from tested null models under stated assumptions, not as proof of a single behavioral mechanism or of deliberate route-oriented placement. We also followed the reviewer’s recommendation to place greater emphasis on effect sizes, sensitivity diagnostics, and model dependence rather than threshold significance. The buffer sweep, geomorphic scenarios, and k-sensitivity analyses are now treated explicitly as sensitivity diagnostics. This has made the argument more cautious, but also more methodologically defensible.

We also agreed with Reviewer 3 that the absence of a full terrain-feasibility model is a major limitation. The revised manuscript now states this directly. It makes clear that the study does not include a bare-earth DTM and does not model slope, curvature, roughness, substrate stability, or other terrain variables directly. The geomorphically screened windows are now explicitly characterized as exploratory rather than as substitutes for terrain-based feasibility modeling. In the same spirit, we responded to the reviewer’s concern about spatial accuracy by adding a dedicated Methods paragraph stating that formal RPAS spatial-accuracy metadata such as GSD, RMSE, and ground-control-point performance are unavailable for the present dataset, and that the analyses should therefore be interpreted at broad spatial scale rather than as high-precision locational tests. We also revised the Discussion to distinguish much more clearly between general movement channeling imposed by quebrada topography and hypothesized association with specific mapped routes. Finally, we agreed fully that the Huarabí ceramics should be treated strictly as evidence of Formative activity nearby, not direct geoglyph dating, and revised the paper accordingly. The reviewer also rightly noted the earlier omission of explicit ethics/permitting information, and we corrected that by adding a dedicated ethics statement specifying the Ministry of Culture resolution number and the authors’ COARPE/RNA status.

In sum, the revision now makes a narrower but more defensible argument. Huarabí provides evidence of Formative-period activity in the vicinity of one geoglyph locality, Pichausa remains chronologically provisional, mapped routes are treated as present-landscape proxies rather than securely dated ancient roads, and the statistical results are interpreted as model-conditional differences from tested null models rather than as definitive evidence of a single cultural mechanism. We believe the manuscript is substantially stronger as a result of the reviewers’ comments, and we are grateful for the opportunity to revise it in this direction.

Sincerely,

Attachment Submitted filename: Letter to reviewers.docx

# Peer review: Decision Letter 1

**DOI:** 10.1371/journal.pone.0350855.r003

**Authors:** Christian Reepmeyer

8 May 2026

PONE-D-26-03379R1

Geoglyphs and Formative-period activity in the middle Chillón Valley, Peru: ceramic association and null-model tests of route proximity

PLOS One

Dear Dr. Mesia-Montenegro,

Thank you for submitting your manuscript to PLOS ONE. After careful consideration, we feel that it has merit but does not fully meet PLOS ONE’s publication criteria as it currently stands. Therefore, we invite you to submit a revised version of the manuscript that addresses the points raised during the review process.

Please submit your revised manuscript by Jun 22 2026 11:59PM. If you will need more time than this to complete your revisions, please reply to this message or contact the journal office at plosone@plos.org. When you're ready to submit your revision, log on to https://www.editorialmanager.com/pone/ and select the 'Submissions Needing Revision' folder to locate your manuscript file.

Please include the following items when submitting your revised manuscript:

- A letter that responds to each point raised by the academic editor and reviewer(s). You should upload this letter as a separate file labeled 'Response to Reviewers'.
- A marked-up copy of your manuscript that highlights changes made to the original version. You should upload this as a separate file labeled 'Revised Manuscript with Track Changes'.
- An unmarked version of your revised paper without tracked changes. You should upload this as a separate file labeled 'Manuscript'.

If you would like to make changes to your financial disclosure, please include your updated statement in your cover letter. Guidelines for resubmitting your figure files are available below the reviewer comments at the end of this letter.

If applicable, we recommend that you deposit your laboratory protocols in protocols.io to enhance the reproducibility of your results. Protocols.io assigns your protocol its own identifier (DOI) so that it can be cited independently in the future. For instructions see: https://journals.plos.org/plosone/s/submission-guidelines#loc-laboratory-protocols. Additionally, PLOS ONE offers an option for publishing peer-reviewed Lab Protocol articles, which describe protocols hosted on protocols.io. Read more information on sharing protocols at https://plos.org/protocols?utm_medium=editorial-email&utm_source=authorletters&utm_campaign=protocols.

As the corresponding author, your ORCID iD is verified in the submission system and will appear in the published article. PLOS supports the use of ORCID, and we encourage all coauthors to register for an ORCID iD and use it as well. Please encourage your coauthors to verify their ORCID iD within the submission system before final acceptance, as unverified ORCID iDs will not appear in the published article. *Only* the individual author can complete the verification step; PLOS staff *cannot* verify ORCID iDs on behalf of authors.

We look forward to receiving your revised manuscript.

Kind regards,

Christian Reepmeyer, PhD

Academic Editor

PLOS One

Journal Requirements:

If the reviewer comments include a recommendation to cite specific previously published works, please review and evaluate these publications to determine whether they are relevant and should be cited. There is no requirement to cite these works unless the editor has indicated otherwise.

Please review your reference list to ensure that it is complete and correct. If you have cited papers that have been retracted, please include the rationale for doing so in the manuscript text, or remove these references and replace them with relevant current references. Any changes to the reference list should be mentioned in the rebuttal letter that accompanies your revised manuscript. If you need to cite a retracted article, indicate the article’s retracted status in the References list and also include a citation and full reference for the retraction notice.

**Additional Editor Comments:**

Please review the comments closely, particularly of Reviewer 2 in reference to the tentative nature of the results and the relative small size of the sample dataset.

[Note: HTML markup is below. Please do not edit.]

Reviewers' comments:

Reviewer's Responses to Questions

**Comments to the Author**

1. If the authors have adequately addressed your comments raised in a previous round of review and you feel that this manuscript is now acceptable for publication, you may indicate that here to bypass the “Comments to the Author” section, enter your conflict of interest statement in the “Confidential to Editor” section, and submit your "Accept" recommendation.

Reviewer #1: All comments have been addressed

Reviewer #3: (No Response)

**********

2. Is the manuscript technically sound, and do the data support the conclusions?

The manuscript must describe a technically sound piece of scientific research with data that supports the conclusions. Experiments must have been conducted rigorously, with appropriate controls, replication, and sample sizes. The conclusions must be drawn appropriately based on the data presented.

Reviewer #1: Yes

Reviewer #3: Partly

**********

3. Has the statistical analysis been performed appropriately and rigorously?

Reviewer #1: I Don't Know

Reviewer #3: Yes

**********

4. Have the authors made all data underlying the findings in their manuscript fully available?

The PLOS Data policy requires authors to make all data underlying the findings described in their manuscript fully available without restriction, with rare exception (please refer to the Data Availability Statement in the manuscript PDF file). The data should be provided as part of the manuscript or its supporting information, or deposited to a public repository. For example, in addition to summary statistics, the data points behind means, medians and variance measures should be available. If there are restrictions on publicly sharing data—e.g. participant privacy or use of data from a third party—those must be specified.

Reviewer #1: Yes

Reviewer #3: Yes

**********

5. Is the manuscript presented in an intelligible fashion and written in standard English?

PLOS ONE does not copyedit accepted manuscripts, so the language in submitted articles must be clear, correct, and unambiguous. Any typographical or grammatical errors should be corrected at revision, so please note any specific errors here.

Reviewer #1: Yes

Reviewer #3: No

**********

6. Review Comments to the Author

Please use the space provided to explain your answers to the questions above. You may also include additional comments for the author, including concerns about dual publication, research ethics, or publication ethics. (Please upload your review as an attachment if it exceeds 20,000 characters)

Reviewer #1: The revised manuscript is much improved, and the authors clearly review their responses to reviewer comments in the cover letter. The overall purpose of the article is clearer, and it now does a nice job of balancing between very careful fine-grained empirical analysis and a broader cultural context that is very cautious about inferences being made, and thoughtful about prospective future directions for the work. There are some minor edits that need to be made with the text (accenting, bolded text where it should not be, etc.), but I think this paper should be accepted for publication.

Reviewer #3: The revised manuscript significantly enhances transparency, interpretive caution, and adherence to ethical standards. The authors have thoughtfully addressed the previous review and have commendably reframed their claims to be model-conditional and exploratory instead of deterministic. However, some key methodological issues, especially in terrain-based feasibility modeling, remain unresolved, limiting the interpretive value of the analysis. Although the manuscript has improved significantly, it still needs specific clarifications and a softer presentation of its conclusions before publication.

The revision significantly enhances the manuscript by clarifying that the analysis is exploratory, model-dependent, and affected by how availability is defined. The authors now avoid interpreting Monte Carlo p-values as direct indicators of intentional or culturally meaningful route placement, instead focusing more on effect sizes, sensitivity analyses, and the variability of results across different windows. This correction is both welcome and important. The limited empirical sample is now more openly acknowledged, and the discussion no longer portrays the results as covering the entire valley or being behaviorally deterministic. However, some language still implies a stronger notion of “structured placement" than the data can support, especially considering the dataset includes only four geoglyph units and six ceramic findspots. The new framing is largely acceptable, but the manuscript could benefit from further subtle adjustments: the results should be uniformly described as deviations from specific tested null models rather than as proof of broader spatial structuring in archaeology. Some phrases continue to suggest a greater degree of spatial organization than the data justifies.

The revision also improves transparency by explicitly acknowledging the lack of terrain-based feasibility modeling, including a bare-earth DTM, slope, substrate, and geomorphic suitability. Adding geomorphic screening and emphasizing that buffer-based availability windows are only geometric proxies for feasible placement areas are valuable clarifications. These updates enhance the methodology's transparency and better align the interpretation with the data's actual limitations. However, the main issue persists: feasibility is still not directly modeled, and the null model assumes spatial availability that may not reflect true construction constraints in quebrada landscapes. Consequently, observed deviations might partly result from misspecification of the counterfactual rather than genuine spatial patterns. Although this limitation is now acknowledged, it would be clearer to explicitly state that the absence of terrain-based feasibility modeling fundamentally restricts the interpretive scope of the analysis and confines the results to exploratory, model-dependent insights.

Regarding spatial accuracy and data resolution, the revision effectively addresses the initial concerns by clearly acknowledging the lack of formal spatial-accuracy metadata, such as ground sampling distance (GSD), georeferencing error, and ground-control-point statistics. The authors smartly reframe their analysis as being conducted at a broad spatial scale and avoid making claims that rely on high positional accuracy. This clarification enhances the credibility of the spatial analysis and ensures the interpretation matches the data's actual resolution. However, the issue remains a limitation, especially since some reported effect sizes occur at relatively short distances where positional uncertainty could be significant. While the manuscript now treats this as a constraint rather than an oversight, it would be improved by explicitly stating that spatial uncertainty might be comparable to some observed differences, emphasizing the exploratory and non-deterministic nature of the findings.

The revision significantly improves the distinction between movement constraints and route association. The authors now clearly differentiate the general movement limitations caused by quebrada topography from the hypothesis of intentional geoglyph placement related to specific circulation routes. This distinction is consistently explained throughout the revised Discussion and is supported by explicit statements noting that proximity to mapped routes alone does not imply intentional association. Clarifying that geomorphological constraints and cultural patterns are analytically distinct enhances the interpretive framework and minimizes the risk of circular reasoning. However, while the conceptual separation is well established, the analysis still lacks independent terrain-based modeling, preventing a clear disentangling of geomorphology's role from potential cultural siting preferences. Thus, the revision addresses the conceptual issue, but empirically separating these factors remains a task for future research.

Regarding chronology and ceramic associations, the revision significantly enhances how the available evidence is addressed by adopting a cautious and appropriately limited interpretive approach. The authors now clearly state that Formative ceramics from Huarabí indicate nearby activity rather than directly dating geoglyph construction. They also openly acknowledge the small sample size, surface context, and potential post-depositional mobility of the artifacts. The difference between Huarabí and Pichausa is now more transparently explained, with Pichausa described as chronologically unresolved rather than implicitly attributed. These corrections are important and necessary. However, some phrasing still suggests that Huarabí provides a "stronger” basis for cultural attribution, which may overstate what limited, ambiguous evidence can support. Overall, the revision could be improved by refining the language to emphasize that ceramics are only useful as contextual indicators of local activity, not as definitive markers of chronological or cultural placement.

Regarding fieldwork authorization and research ethics, the revised manuscript fully addresses this concern. It ensures the manuscript complies with Peru's archaeological research standards and enhances its transparency and credibility. Including this information was necessary and is presented clearly and professionally.

Regarding the conceptual contribution and interpretive framing, the revision marks a notable improvement in caution, transparency, and alignment between data and claims. The manuscript now regularly emphasizes that its findings are model-dependent and stops short of claiming definitive evidence of culturally meaningful spatial patterns. The shift to highlight exploratory analysis, conditional inference, and the constraints of data quality and sample size is fitting and bolsters the overall argument. Additionally, the discussion becomes more constructive by placing the geoglyphs within a wider landscape context, including movement, encounter, and possible ritual activities, rather than concentrating solely on route proximity. Nevertheless, the interpretive contribution remains somewhat limited, mainly because empirical constraints (such as small sample size, weak chronological control, and lack of terrain-based feasibility modeling) limit the strength of cultural inferences. Although these limitations are now acknowledged, framing the main contribution more explicitly as methodological and exploratory, rather than as offering a substantive cultural explanation, would enhance the manuscript.

**********

7. PLOS authors have the option to publish the peer review history of their article (what does this mean?). If published, this will include your full peer review and any attached files.

If you choose “no”, your identity will remain anonymous but your review may still be made public.

**Do you want your identity to be public for this peer review?** For information about this choice, including consent withdrawal, please see our Privacy Policy.

Reviewer #1: No

Reviewer #3: No

**********

[NOTE: If reviewer comments were submitted as an attachment file, they will be attached to this email and accessible via the submission site. Please log into your account, locate the manuscript record, and check for the action link "View Attachments". If this link does not appear, there are no attachment files.]

To ensure your figures meet our technical requirements, please review our figure guidelines: https://journals.plos.org/plosone/s/figures

You may also use PLOS’s free figure tool, NAAS, to help you prepare publication quality figures: https://journals.plos.org/plosone/s/figures#loc-tools-for-figure-preparation.

NAAS will assess whether your figures meet our technical requirements by comparing each figure against our figure specifications.

# Author comment: Author response to Decision Letter 2

**DOI:** 10.1371/journal.pone.0350855.r004

13 May 2026

Lima, May 8th, 2026

Dear Dr. Reepmeyer,

Academic Editor

PLOS ONE

We thank you and the reviewers for the careful assessment of our revised manuscript. We have made a further revision focused on the remaining issues raised in this round: the tentative character of the results, the small empirical sample, the limits of the availability model, and the need to present the contribution as exploratory and methodological rather than as a substantive cultural explanation.

The manuscript now frames the results consistently as deviations from specified null models, not as evidence of intentional route association, construction chronology, or valley-wide spatial organization. We also clarify that the Huarabí ceramics indicate nearby Formative-period activity but do not directly date the geoglyphs, and that Pichausa remains chronologically unresolved. Additional revisions address spatial uncertainty, route proxies, survey-effort uncertainty, and the absence of terrain-based construction-feasibility modeling.

Our responses are provided below.

Response to the Academic Editor

Comment:

“Please review the comments closely, particularly of Reviewer 2 [Reviewer 3 in the comments below] in reference to the tentative nature of the results and the relative small size of the sample dataset.”

Response:

We have revised the manuscript to make the tentative scope of the analysis explicit. The Abstract now states the empirical scale of the study—four geoglyph groups and six Huarabí ceramic findspots—and specifies that the results do not establish intentional route association, construction age, or valley-wide spatial organization. The Conclusions have also been revised to state that the findings should be read as exploratory, model-dependent evidence of deviation from tested null models.

We strengthened the limitations section by emphasizing the small sample, the restriction of inference to the recorded contexts, and the fact that the analysis should not be generalized to the entire valley. We also added clearer language explaining that the availability windows are geometric proxies rather than terrain-based models of construction feasibility.

General comment

Reviewer 3:

“The revised manuscript significantly enhances transparency, interpretive caution, and adherence to ethical standards. The authors have thoughtfully addressed the previous review and have commendably reframed their claims to be model-conditional and exploratory instead of deterministic. However, some key methodological issues, especially in terrain-based feasibility modeling, remain unresolved, limiting the interpretive value of the analysis. Although the manuscript has improved significantly, it still needs specific clarifications and a softer presentation of its conclusions before publication.”

Response:

We thank Reviewer 3 for recognizing the improvements in transparency, interpretive caution, and ethical reporting. We agree that the absence of terrain-based feasibility modeling limits the interpretive scope of the analysis. We have therefore revised the Abstract, Methods, Discussion, and Conclusions to make this limitation more explicit.

The revised manuscript now states that the availability windows are geometric approximations rather than independently modeled construction-feasibility surfaces. It also specifies that observed deviations may reflect cultural siting preferences, geomorphic constraints, visibility conditions, survey-process effects, or misspecification of the counterfactual placement space. The Conclusions have been softened accordingly.

Model-dependent inference and “structured placement”

Reviewer 3:

“Some language still implies a stronger notion of ‘structured placement’ than the data can support, especially considering the dataset includes only four geoglyph units and six ceramic findspots. The results should be uniformly described as deviations from specific tested null models rather than as proof of broader spatial structuring in archaeology.”

Response:

We have removed language that could be read as claiming broader spatial structuring. The manuscript now describes the results as deviations from tested null models under stated availability assumptions. Terms such as “structured placement” have been replaced with more precise language, including “model-dependent spatial discrepancy,” “deviation from the tested null model,” and “difference between observed and simulated distance distributions.”

The Discussion now states that the results support only a model-dependent spatial discrepancy in Huarabí, not intentional route association or broader spatial organization. The Conclusions likewise state that the findings are exploratory and model-dependent, rather than evidence for a single cultural mechanism.

Terrain-based feasibility modeling

Reviewer 3:

“The main issue persists: feasibility is still not directly modeled, and the null model assumes spatial availability that may not reflect true construction constraints in quebrada landscapes. Consequently, observed deviations might partly result from misspecification of the counterfactual rather than genuine spatial patterns.”

Response:

We have expanded this limitation in the Methods. The manuscript now states that the availability windows are geometric approximations of potential placement space rather than independently modeled construction-feasibility surfaces. We specify that the analysis does not incorporate bare-earth DTM-derived slope, microtopography, substrate, surface clast density, visibility of cleared lines, erosion history, or geomorphic stability.

We did not add a new terrain-based feasibility model because the required bare-earth terrain and substrate data are not available for the present dataset. Instead, we have restricted the interpretation and identify terrain-based feasibility modeling as a priority for future work.

Spatial accuracy and uncertainty

Reviewer 3:

“Spatial uncertainty might be comparable to some observed differences, emphasizing the exploratory and non-deterministic nature of the findings.”

Response:

We added explicit language on spatial uncertainty. The Methods now state that formal spatial-accuracy metadata, including ground sampling distance, georeferencing RMSE, and ground-control-point error statistics, are unavailable for the present dataset. We also note that, because some reported contrasts occur at relatively short distance scales, positional uncertainty may be non-trivial relative to the smaller observed differences.

This clarification reinforces the treatment of the analysis as a broad-scale spatial comparison, not a high-precision test of exact locational accuracy.

Movement constraints versus route association

Reviewer 3:

“The revision significantly improves the distinction between movement constraints and route association. However, while the conceptual separation is well established, the analysis still lacks independent terrain-based modeling, preventing a clear disentangling of geomorphology’s role from potential cultural siting preferences.”

Response:

We have retained the distinction between general movement constraints imposed by quebrada topography and proximity to mapped route proxies. We also added stronger caution that these factors cannot be separated with the present data.

The Discussion now states that apparent route proximity may partly reflect geomorphological channeling and survey process rather than intentional siting relative to circulation corridors. The future-work section identifies terrain-based feasibility modeling, visibility analysis, and standardized route representations as necessary steps for distinguishing cultural siting practices from geomorphic constraints and survey effects.

Chronology and ceramic associations

Reviewer 3:

“The revision could be improved by refining the language to emphasize that ceramics are only useful as contextual indicators of local activity, not as definitive markers of chronological or cultural placement.”

Response:

We revised the treatment of the ceramic evidence throughout the manuscript. The Abstract now states that diagnostic Formative-period ceramics were recorded only in Huarabí, where they indicate nearby activity rather than directly dating geoglyph construction. The ceramic-analysis section treats ceramics as a coarse typological attribution based on surface material, not as a basis for fine-grained phasing or direct dating.

The Discussion now states that Huarabí provides the only currently documented contextual indication of nearby Formative-period activity, and that the depositional relationship between the sherds and the linework cannot be established. We also clarify that Pichausa is not assigned a secure Formative chronology; its irregular morphology is treated only as a comparative observation.

Conceptual contribution and interpretive framing

Reviewer 3:

“Framing the main contribution more explicitly as methodological and exploratory, rather than as offering a substantive cultural explanation, would enhance the manuscript.”

Response:

We have revised the framing accordingly. The Abstract now identifies the principal contribution as methodological: the study demonstrates how small geoglyph datasets can be evaluated through transparent null-model procedures while making explicit the limits imposed by sample size, surface chronology, spatial uncertainty, and the absence of terrain-based feasibility modeling.

The Discussion now treats the Huarabí result as a basis for further testing rather than as a substantive explanation. The final future-work paragraph has also been revised to state that future research should test whether the observed proximity patterns reflect cultural siting practices, geomorphic constraints, visibility conditions, survey-process effects, or some combination of these factors.

We thank you and both reviewers for their detailed comments. The revised manuscript now presents the analysis as exploratory, model-dependent, and methodologically focused. It avoids stronger cultural claims that are not supported by the present sample and clarifies the limits imposed by surface chronology, spatial uncertainty, route proxies, survey-effort uncertainty, and the absence of terrain-based feasibility modeling.

Sincerely,

Christian Mesía-Montenegro & Angel Sánchez-Borjas

Attachment Submitted filename: Letter to reviewers v.2.docx

# Editor report: Decision Letter 2

**DOI:** 10.1371/journal.pone.0350855.r005

**Authors:** Christian Reepmeyer

19 May 2026

Geoglyphs and Formative-period activity in the middle Chillón Valley, Peru: ceramic association and null-model tests of route proximity

PONE-D-26-03379R2

Dear Dr. Mesia-Montenegro,

We’re pleased to inform you that your manuscript has been judged scientifically suitable for publication and will be formally accepted for publication once it meets all outstanding technical requirements.

Within one week, you’ll receive an e-mail detailing the required amendments. When these have been addressed, you’ll receive a formal acceptance letter and your manuscript will be scheduled for publication.

An invoice will be generated when your article is formally accepted. Please note, if your institution has a publishing partnership with PLOS and your article meets the relevant criteria, all or part of your publication costs will be covered. Please make sure your user information is up-to-date by logging into Editorial Manager at Editorial Manager® and clicking the ‘Update My Information' link at the top of the page. For questions related to billing, please contact billing support.

If your institution or institutions have a press office, please notify them about your upcoming paper to help maximize its impact. If they’ll be preparing press materials, please inform our press team as soon as possible -- no later than 48 hours after receiving the formal acceptance. Your manuscript will remain under strict press embargo until 2 pm Eastern Time on the date of publication. For more information, please contact onepress@plos.org.

Kind regards,

Christian Reepmeyer, PhD

Academic Editor

PLOS One

Additional Editor Comments (optional):

Reviewers' comments:

# Editor report: Acceptance letter

**DOI:** 10.1371/journal.pone.0350855.r006

**Authors:** Christian Reepmeyer

PONE-D-26-03379R2

PLOS One

Dear Dr. Mesia-Montenegro,

I'm pleased to inform you that your manuscript has been deemed suitable for publication in PLOS One. Congratulations! Your manuscript is now being handed over to our production team.

At this stage, our production department will prepare your paper for publication. This includes ensuring the following:

* All references, tables, and figures are properly cited

* All relevant supporting information is included in the manuscript submission,

* There are no issues that prevent the paper from being properly typeset

You will receive further instructions from the production team, including instructions on how to review your proof when it is ready. Please keep in mind that we are working through a large volume of accepted articles, so please give us a few days to review your paper and let you know the next and final steps.

Lastly, if your institution or institutions have a press office, please let them know about your upcoming paper now to help maximize its impact. If they'll be preparing press materials, please inform our press team within the next 48 hours. Your manuscript will remain under strict press embargo until 2 pm Eastern Time on the date of publication. For more information, please contact onepress@plos.org.

You will receive an invoice from PLOS for your publication fee after your manuscript has reached the completed accept phase. If you receive an email requesting payment before acceptance or for any other service, this may be a phishing scheme. Learn how to identify phishing emails and protect your accounts at https://explore.plos.org/phishing.

If we can help with anything else, please email us at customercare@plos.org.

Thank you for submitting your work to PLOS ONE and supporting open access.

Kind regards,

PLOS ONE Editorial Office Staff

on behalf of

Dr. Christian Reepmeyer

Academic Editor

PLOS One
